# Gut microbial‐derived 3,4‐dihydroxyphenylacetic acid ameliorates reproductive phenotype of polycystic ovary syndrome

**DOI:** 10.1002/imt2.70065

**Published:** 2025-07-15

**Authors:** Pan Li, Li Xie, Huimin Zheng, Yinglin Feng, Feihong Mai, Wenli Tang, Jiajia Wang, Zixin Lan, Shuaijun Lv, Thisun Jayawardana, Sabrina Koentgen, Shuangbin Xu, Zhengwei Wan, Yunjie Chen, Haiyan Xu, Sj Shen, Fan Zhang, Yuanhao Yang, Georgina Hold, Fangjie He, Emad M. El‐Omar, Guangchuang Yu, Xia Chen

**Affiliations:** ^1^ Ningbo Key Laboratory of Human Microbiome and Precision Medicine, Central Laboratory of the Medical Research Center The First Affiliated Hospital of Ningbo University Ningbo China; ^2^ Department of Obstetrics and Gynecology The First Affiliated Hospital of Ningbo University Ningbo China; ^3^ UNSW Microbiome Research Centre St George and Sutherland Clinical Campuses, UNSW Sydney Australia; ^4^ Department of Obstetrics, Affiliated Foshan Maternity & Child Healthcare Hospital Southern Medical University Foshan China; ^5^ Institute of Ecological Science, School of Life Science South China Normal University Guangzhou China; ^6^ The Second Clinical Medical College Southern Medical University Guangzhou China; ^7^ Department of Bioinformatics, School of Basic Medical Sciences Southern Medical University Guangzhou China; ^8^ Department of Health Management & Institute of Health Management, Sichuan Provincial People's Hospital University of Electronic Science and Technology of China Chengdu China; ^9^ Department of Pharmacy The First Affiliated Hospital of Ningbo University Ningbo China; ^10^ Reproductive Medicine Center The First Affiliated Hospital of Ningbo University Ningbo China; ^11^ Mater Research Institute The University of Queensland Woolloongabba Queensland Australia; ^12^ Fujian Maternity and Child Health Hospital, College of Clinical Medicine for Obstetrics & Gynecology and Pediatrics Fujian Medical University Fuzhou China

**Keywords:** 3,4‐dihydroxyphenylacetic acid, gut metabolome, gut microbiota, polycystic ovary syndrome, *Streptococcus thermophilus*, β‐galactosidase

## Abstract

Polycystic ovary syndrome (PCOS) is a prevalent endocrine and reproductive disorder affecting women of reproductive age. While the gut microbiota has been implicated in PCOS pathophysiology, the role of microbial‐derived metabolites as mediators of host–microbe interactions remains poorly defined. Here, we integrated untargeted gut metabolomics with metagenomic profiling in patients with PCOS and identified a marked depletion of 3,4‐dihydroxyphenylacetic acid (DHPAA), a flavonoid‐derived microbial catabolite. Oral administration of DHPAA ameliorated PCOS‐like phenotypes in two mouse models by suppressing bone morphogenetic protein signaling and reducing anti‐Müllerian hormone (AMH) levels. We found that DHPAA production depends on gut microbial degradation of dietary flavonoids. We further identified a bacterial species, *Streptococcus thermophilus*, consistently depleted in PCOS across two human cohorts and a mouse model, restored DHPAA levels and improved reproductive outcomes in mice. Conversely, a β‐galactosidase‐deficient mutant of *S. thermophilus* failed to confer these benefits, highlighting β‐galactosidase as a critical enzyme in DHPAA biosynthesis. Our findings establish DHPAA as a key microbial metabolite linking diet, microbiota, and reproductive health, and propose its potential as a novel therapeutic candidate for PCOS.

## Highlights

- 3,4‐dihydroxyphenylacetic acid (DHPAA) is identified as a key microbial‐derived metabolite responsible for the protective effects of flavonoids against polycystic ovary syndrome (PCOS).
- *Streptococcus thermophilus* produces DHPAA from flavonoids through β‐galactosidase‐mediated metabolism.
- Gut microbiota is essential for transforming flavonoids into bioactive metabolites that confer protection against PCOS.

## Graphical abstract

By integrating multi‐level and multi‐omics analyses, we identify 3,4‐dihydroxyphenylacetic acid (DHPAA), a gut microbiota‐derived degradation product of dietary flavonoids, as a key bioactive end‐product responsible for the beneficial effects against polycystic ovary syndrome (PCOS). DHPAA exerts direct therapeutic effects on PCOS‐like phenotypes independent of the gut microbiota through inhibition of bone morphogenetic protein signaling. We further demonstrate that *Streptococcus thermophilus* metabolizes flavonoids into DHPAA via β‐galactosidase activity, thereby contributing to the improvement of PCOS symptoms. These findings highlight the essential role of the gut microbiota in transforming flavonoids into functionally active metabolites and establish DHPAA as a critical mediator linking microbial metabolism to host reproductive health. This study deepens our understanding of the microbiota‐metabolite‐host axis in PCOS pathophysiology and provides a promising candidate molecule for therapeutic development.

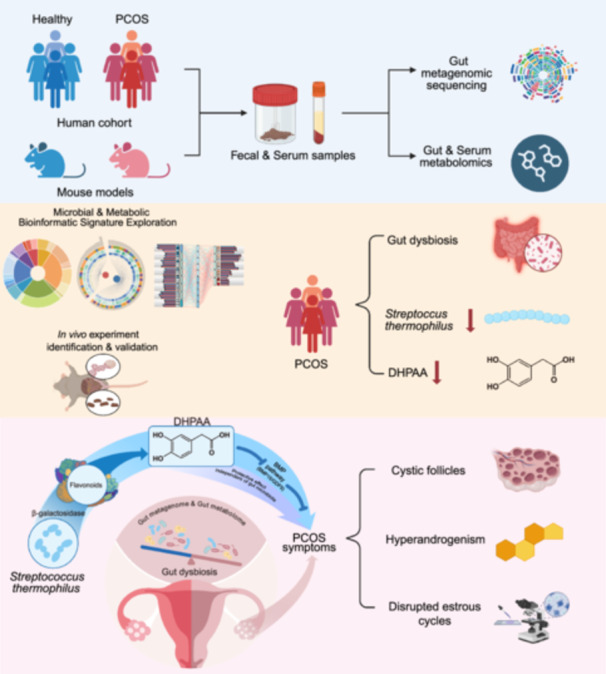

## INTRODUCTION

Polycystic ovary syndrome (PCOS), characterized by hyperandrogenism, ovulatory dysfunction, and polycystic ovarian morphology, is one of the most common disorders in women. PCOS affects 10%–13% of women of reproductive age [1, 2] and is often accompanied by an increased risk of metabolic and psychological conditions [3], imposing a significant burden on both affected individuals and society [4]. However, the insufficient knowledge of the pathogenesis of PCOS results in the current reliance on symptom‐based treatment [5]. The gaps in understanding the etiology of PCOS hinder the development of mechanism‐based targeted therapies, emphasizing the need to elucidate its pathogenesis and seek disease‐specific treatments.

Recent research has revealed that gut microbiota and its metabolites contribute significantly to the pathogenesis of PCOS [6, 7, 8, 9]. In 2019, a milestone study by Qi and colleagues demonstrated that gut bacteria transplantation can induce PCOS‐like symptoms in mice, suggesting a potential causal role for gut microbiota in PCOS [7]. Additionally, their studies highlighted the significant contribution of bacterial‐derived metabolites to the development of PCOS [7, 9]. Furthermore, the protective effects of modulating gut microbiota, such as lowering testosterone levels and improving ovarian morphology, through interventions like probiotics and metabolite supplements [7, 9, 10, 11, 12] suggest promising avenues for novel treatments. Thus, exploring the relationship between microbial communities, metabolites, and their interplay with the host can deepen our understanding of the pathogenesis of PCOS and uncover novel therapeutic targets. However, there is still a lack of untargeted metabolomics studies investigating the gut metabolite profile of PCOS, which could identify both potential therapeutic and pathogenic metabolites. Therefore, although previous studies identified potential pathogenic bacteria and metabolites [7, 9], the identification of potential therapeutic, their derivation from gut bacteria, and their effects on the host remain to be studied.

To address the questions above, we performed an integrative analysis of metabolomics and metagenomics in patients with PCOS and controls to investigate the gut metabolite profile of PCOS and its interaction with gut microbiota. A potential therapeutic metabolite, 3,4‐dihydroxyphenylacetic acid (DHPAA), was identified and verified in mouse experiments. Gut microbiota significantly influenced the concentrations of DHPAA in both feces and serum, and was involved in its production. Following this, we identified the decrease of *Streptococcus thermophilus* in PCOS by integrating gut metagenomic analyses in two human cohorts and a mouse model, and we further verified its capability of producing DHPAA via β‐galactosidase. Collectively, we profiled the gut metabolome of patients with PCOS, and our findings provide novel candidates to mitigate PCOS.

## RESULT

### Gut metabolome analysis reveals distinct metabolite pattern in PCOS

**FIGURE 1:**
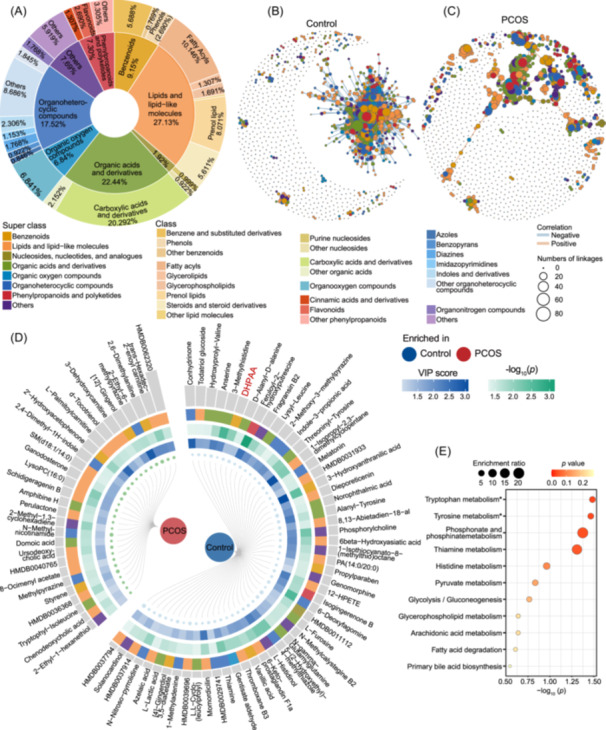
Gut metabolomic profiles of patients with polycystic ovary syndrome (PCOS). (A) Composition of the identified metabolites; the inner cycle indicates the super class of metabolites, and the outer cycle indicates the class of metabolites. (B and C) The interaction networks of metabolites in healthy controls (left, B) and patients with PCOS (right, C). Each node represents a metabolite colored by super class. Each edge represents a significant correlation between the pairs of metabolites. (D) Results indicated 81 differential metabolites between patients with PCOS and healthy controls. The height of bars indicates fold change between groups, the blue cube indicates variable importance in projection (VIP) scores of metabolites, and the green cube indicates the *p*‐value using two‐tailed Wilcoxon rank‐sum test. The enriched/decreased metabolites were defined by orthogonal partial least squares discriminant analysis (OPLS‐DA) VIP score > 1 and two‐tailed Wilcoxon rank‐sum test *p*‐value < 0.05. (E) Pathway enrichment analysis of the differential metabolites between patients with PCOS and healthy controls.

To investigate the gut metabolomic profiles of PCOS, we employed untargeted gut metabolomics by liquid chromatography‐mass spectrometry using the fecal samples of 28 healthy controls and 30 patients with PCOS (Table S1). A total of 1301 metabolites belonging to 125 classes and 19 super classes were identified. Among these identified metabolites, the gut metabolites of patients with PCOS were composed of the super class of lipids and lipid‐like molecules, organic acids and derivatives, organoheterocyclic compounds, phenylpropanoids and polyketides, and benzenoids (Figure 1A). We then compared the overall differences of gut metabolome between patients with PCOS and healthy controls. Although no significant compositional difference of gut metabolome had been found (Figure S1A,B), co‐occurrence network analyses revealed a distinct metabolite correlative pattern between patients with PCOS and healthy controls (Figure 1B,C). Overall, when compared with the healthy controls, patients with PCOS had a marked reduction in connections of the interaction network (Table S2) and a significantly higher variation of metabolites (Figure S1C). We observed a shift in the coefficient of variation when comparing all metabolites between PCOS group and control group (Figure S1D). Metabolites from the super class of flavonoids, steroids and steroid derivatives, and pyridines and derivatives exhibited high coefficients of variation (CV, Figure S1E). We summarized the distribution of metabolites with higher CVs in each group. We found that 778 metabolites had higher CVs in PCOS patients, whereas only 513 metabolites had higher CVs in the control group. Additionally, we applied the Modified Signed‐Likelihood Ratio Test (MSLRT) for equality of CVs and observed that 58 metabolites had significantly higher CVs in PCOS patients, compared to 36 in the control group (Table S3). Next, we further investigate the difference in specific metabolites to seek for disease‐related biomarkers by orthogonal partial least squares discriminant analysis and Wilcoxon rank‐sum test. There were 28 enriched and 53 decreased metabolites in the PCOS group when compared with the control group, mainly belong to the super class of benzenoids, lipids and lipid‐like molecules, organic acids and derivatives, and organoheterocyclic compounds (Figure 1D). Pathway enrichment analysis suggested that these differential metabolites involved in multiple functions, including tryptophan metabolism and tyrosine metabolism (Figure 1E).

### Supplementation of a gut metabolite, DHPAA, prevents PCOS‐like symptoms

Among the metabolites showing significant differences between the two groups, one particular metabolite, DHPAA, a downstream catabolite of flavonoids reported to mitigate multiple diseases [13], caught our attention. We employed a bioinformatic analytic strategy described in our previous study to predict the function of DHPAA and search for its potential association with PCOS [14]. DHPAA was implicated in the reproductive‐related functions, including steroid biosynthetic process, female gonad development steroid metabolism, and hormone ligand‐binding receptors. Disease ontology enrichment analysis indicated that DHPAA might be related to ovarian infertility. The analytic branches of structural similar metabolites and co‐abundant metabolites suggested that DHPAA was associated with female reproductive system diseases and metabolic diseases, including PCOS, ovarian dysfunction, pre‐eclampsia, and fatty liver disease (Figure S2A,B). Then, we verified the concentration of DHPAA in the fecal and serum samples from patients with PCOS and healthy controls using targeted liquid chromatography‐tandem mass spectrometry analysis, finding significantly lower levels of DHPAA in the samples from patients with PCOS (Figure S2C,D). Subsequently, we utilized a well‐established mouse model of PCOS through subcutaneous injection of dehydroepiandrosterone (DHEA) [7, 15]. Consistently, we found that the concentration of DHPAA decreased in the cecum and serum samples of the PCOS‐like mice by targeted liquid chromatography‐tandem mass spectrometry analysis (Figure S2E,F), echoing our results in the human cohort.

**FIGURE 2:**
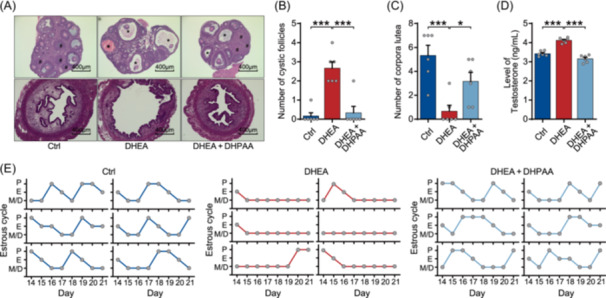
3,4‐dihydroxyphenylacetic acid (DHPAA) alleviates polycystic ovary syndrome (PCOS)‐like symptoms in mice. (A) Hematoxylin and eosin (H&E) staining of representative mouse ovaries and uterus from control mice (Ctrl), PCOS‐like mice (DHEA), and PCOS‐like mice with DHPAA treatment (DHEA + DHPAA). The corpora lutea are indicated by #, and the cystic follicle is indicated by *. (B) Quantitative analysis of cystic follicles in the ovaries (*n* = 6 mice per group). (C) Quantitative analysis of corpora lutea in the ovaries (*n* = 6 mice per group). (D) Levels of testosterone in serum (*n* = 6 mice per group). (E) Estrous cycle analysis (*n* = 6 mice per group). The dose of DHPAA is 80 μM. Data are presented as the mean ± SEM; *p < 0.05, ***p < 0.001 according to two‐tailed one‐way ANOVA followed by Dunnett post hoc test.

To investigate whether DHPAA possesses protective effects against PCOS, oral DHPAA supplementation was applied to PCOS‐like mice (Figure S2G,H). In PCOS‐like mice, the ovaries exhibited an increased number of cyst‐like follicles and a decreased presence of post‐ovulation corpus luteum. In contrast, the ovaries and uterus of DHPAA‐treated mice displayed normal morphology, with ovarian follicles at various stages of development (80 μM DHPAA dissolved in drinking water, Figure 2A–C). Additionally, DHPAA reduced the elevated serum testosterone levels in DHEA‐treated mice, preventing the development of PCOS‐like traits (Figure 2D), and improved the estrous cycle disrupted by DHEA (Figure 2E). The findings were further validated in an additional PCOS‐like mice model induced by letrozole (Figure S3A,B), which is another robust inducer of androgen. The therapeutic efficacy of DHPAA across two PCOS models supports the consistency and reliability of the findings. DHPAA treatment effectively inhibited polycystic ovarian morphology and increased the presence of ovulatory corpora lutea (Figure S3C–E). Additionally, it significantly reduced serum testosterone levels in PCOS‐like mice (Figure S3F) and restored normal estrous cycles (Figure S3H).

Taken together, these results indicate that the significantly decreased level of DHPAA in PCOS may be related to PCOS, and DHPAA can prevent PCOS‐like phenotypes in mice.

### Protective effects of DHPAA against PCOS relate to the inhibition of BMP signaling

**FIGURE 3:**
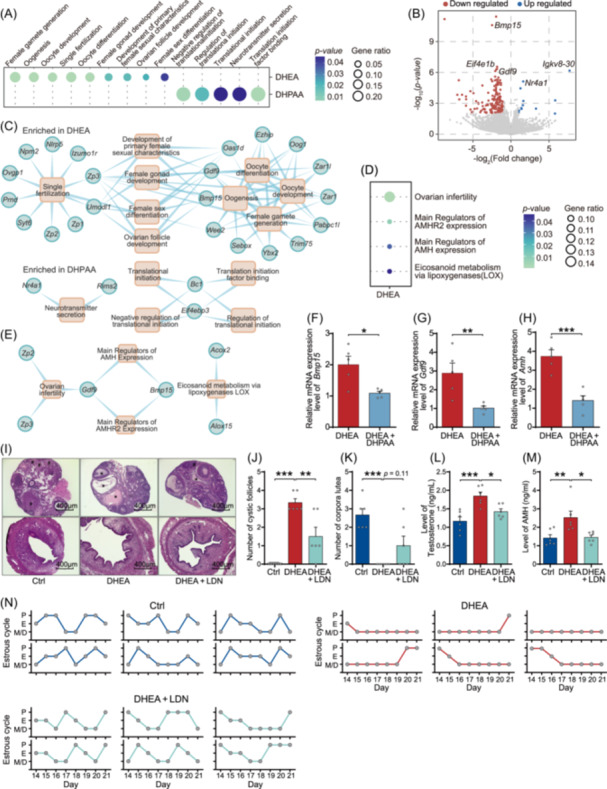
Protective effects of 3,4‐dihydroxyphenylacetic acid (DHPAA) against polycystic ovary syndrome (PCOS) links to inhibiting BMP signaling. (A) Gene ontology analysis of the differential genes between PCOS‐like mice with or without DHPAA pretreatment from ovarian tissue. (B) Volcano plots of the differentially expressed genes. Data were compared using DeSeq. 2. (C) Gene‐concept network analysis of the association between genes and involved pathways based on GO database. (D) Gene ontology analysis of the differentially expressed genes between PCOS‐like mice and control mice based on Wiki pathway database and manual curated database. (E) Gene‐concept network analysis of the association between genes and involved pathways based on Wiki pathway database and manually curated database. (F–H) Expression levels of *Bmp15*, *Gdf9*, and *Amh* in ovary (*n* = 5 mice per group). (I) H&E staining of representative mouse ovaries and uterus from control mice, PCOS‐like mice treated with or without LDN193189 (scale bars, 400 μm). The corpora lutea are indicated by #, and the cystic follicle is indicated by *. (J) Quantitative analysis of cystic follicles in the ovaries (*n* = 6 mice per group). (K) Quantitative analysis of corpora lutea in the ovaries (*n* = 6 mice per group). (L) Levels of testosterone in serum (*n* = 6 mice per group). (M) Levels of AMH in serum (*n* = 6 mice per group). (N) Estrous cycle analysis (*n* = 6 mice per group). The dose of DHPAA is 80 μM. (F–H, J–M) Data are presented as the mean ± SEM; *p < 0.05, **p < 0.01, ***p < 0.001 according to two‐tailed one‐way ANOVA followed by Dunnett post hoc test or two‐tailed Student's *t*‐test. GO, gene ontology.

Next, to explore the mechanism by which DHPAA prevents PCOS‐like phenotype, transcriptomic sequencing was performed on ovarian tissues harvested from PCOS‐like mice with or without DHPAA pretreatment. A significantly different gene expression profile was observed between ovarian tissues from DHPAA‐treated and untreated Cis‐POI mice, and gene ontology analysis showed that the involved pathways were mainly associated with female gonadal development and ovarian follicle development (Figure 3A,B). In the gene‐concept network showing the association between genes and involved pathways based on GO database, we observed that *Bmp15* and *Gdf9*, which are specifically expressed by oocytes at all stages of development [16], were associated with oocyte‐relative pathways (Figure 3C). Previous studies reported that BMP15 can simultaneously upregulate the expression of AMH and AMHR2, and this effect can be enhanced by GDF9 [16, 17, 18]. Considering that overexpression and increased activity of AMH are strongly associated with PCOS [19, 20], these results indicated that the protective effects of DHPAA against PCOS may be related to modulation of BMP signaling and AMH. To further explore the involvement of differentially expressed genes (DEGs) in AMH pathways, we employed Wiki pathway enrichment analysis using clusterProfiler based on the AMH‐related genes [16]. Our analyses showed that both *Bmp15* and *Gdf9* were associated with the regulation of AHM and AMHR2 expression among significantly DEGs between the two groups (Figure 3D,E). Subsequently, we tested the expression levels of these AMH‐related genes by quantitative reverse‐transcription PCR and found significantly lower expression of *Bmp15*, *Gdf9*, and *Amh* in the ovarian tissue of DHPAA pretreated PCOS‐like mice (Figure 3F–H, Figure S4A–F).

To determine the role of BMP signaling, we intravenously injected LDN193189, a selective BMP signaling inhibitor, into PCOS mouse model (Figure S4G,H). As expected, ovarian histological analysis revealed that LDN193189 reversed the PCOS‐like phenotypes in DHEA‐treated mice, as indicated by normalized ovary and uterus morphology (Figure 3I), a decrease in cyst‐like follicles (Figure 3J), and an increase in post‐ovulation corpora lutea (Figure 3K). PCOS‐like mice treated with LDN193189 had significantly serum testosterone levels and reduced *Amh* expression (Figure 3L,M), as well as improved estrous cycles (Figure 3N). In contrast, coadministration of DHPAA and FK506, an agonist of BMP signaling, failed to alleviate PCOS‐like symptoms in mice (Figure S5). Consistently, AMH expression was also decreased in PCOS‐like mice treated with DHPAA alone (Figure S2I, Figure 3G). Taken together, these results indicate that DHPAA alleviates PCOS symptoms by inhibiting the activity of BMP signaling.

### DHPAA is a gut microbial end‐product of flavonoid metabolism

**FIGURE 4:**
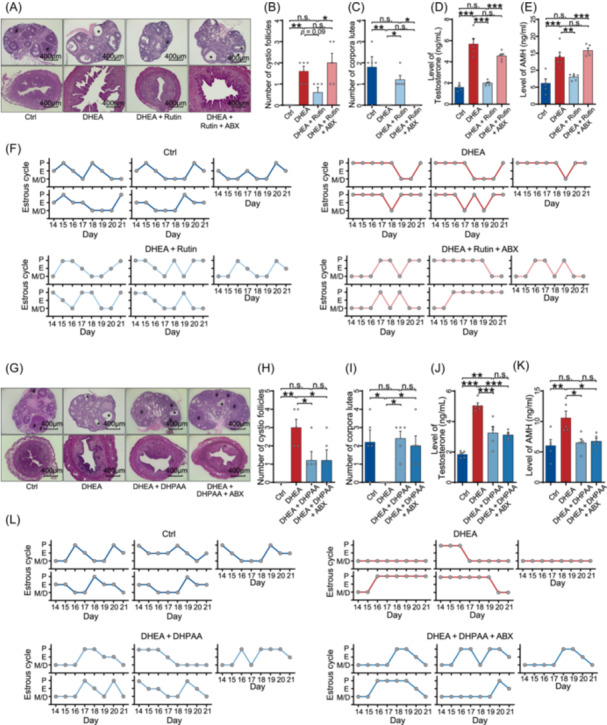
Protective effects of 3,4‐dihydroxyphenylacetic acid (DHPAA) against polycystic ovary syndrome (PCOS) are dependent on the presence of gut microbiota. (A) Representative H&E‐stained sections of ovaries and uterus from control mice, PCOS‐like mice, PCOS‐like mice treated with rutin, and antibiotics‐pretreated PCOS‐like mice treated with rutin (scale bars, 400 μm). Corpora lutea are indicated by #, and cystic follicle is indicated by *. (B) Quantitative analysis of cystic follicles in the ovaries (*n* = 5 mice per group). (C) Quantification of corpora lutea in the ovaries (*n* = 5 mice per group). (D) Levels of testosterone in serum (*n* = 5 mice per group). (E) Levels of AMH in serum (*n* = 5 mice per group). (F) Estrous cycle analysis (*n* = 5 mice per group). (G) Representative H&E‐stained sections of ovaries and uteri from control mice, PCOS‐like mice, PCOS‐like mice treated with DHPAA, and antibiotics‐pretreated PCOS‐like mice treated with DHPAA (scale bars, 400 μm). Corpora lutea are indicated by #, and cystic follicle is indicated by *. (H) Quantification of cystic follicles in the ovaries (*n* = 5 mice per group). (I) Quantitative analysis of corpora lutea in the ovaries (*n* = 5 mice per group). (J) Levels of testosterone in serum (*n* = 5 mice per group). (K) Levels of AMH in serum (*n* = 5 mice per group). (L) Estrous cycle analysis (*n* = 5 mice per group). DHPAA was administered at 80 μM; rutin was administered at 50 μM. Data are presented as the mean ± SEM; *p < 0.05, **p < 0.01, ***p < 0.001 according to two‐tailed one‐way ANOVA followed by Tukey multiple comparison test.

Previous studies have identified DHPAA as one of the most abundant catabolites of flavonoids degraded by gut microbiota [13]. We first tested the cecum and serum samples from mice with and without antibiotics and found that DHPAA levels were significantly lower when antibiotics were applied (Figure S6A,B). Consistent with previous studies, these findings suggest that the level of DHPAA depends on the activities of the gut microbiota. Moreover, rutin, the most widely distributed and abundant flavonoid in food [21, 22], such as apples, berries, and onions, can be metabolized by gut microbiota to produce phenolic derivatives [13], including DHPAA, which is among the most abundant products [23, 24]. We used rutin as a substrate to investigate the role of gut microbiota in mediating the protective effect of DHPAA. Before DHEA injection and rutin administration, we used broad‐spectrum antibiotics to deplete gut microbiota in mice. We first confirmed that antibiotic treatment did not interfere with the establishment of the DHEA‐induced PCOS‐like phenotype (Figure S6C–J), which was consistent with previous findings [25]. Rutin was then administered to PCOS‐like mice with or without antibiotic treatment (Figure S7A,B). We observed a significantly decreased concentration of DHPAA in the cecum and serum samples from PCOS‐like mice treated with antibiotics (Figure S7C,D). Consistent with the concentration change of DHPAA, in mice without antibiotics, rutin pretreatment prevented the PCOS‐like syndromes, including normalized morphology of ovary and uterus, regular estrous cycles, and testosterone secretion, decreased cyst‐like follicles, increased post‐ovulation corpora lutea. It also resulted in fewer cyst‐like follicles, more post‐ovulation corpora lutea, and lower AMH expression in ovarian tissue (Figure 4A–F). However, these protective effects were not observed in PCOS‐like mice treated with antibiotics (Figure 4A–F). Next, we examined the ability of gut microbiota between patients with PCOS and healthy controls in degrading rutin to DHPAA. By culturing fecal samples from both groups with rutin, we found that DHPAA levels were significantly lower in the PCOS group compared to the control group, while rutin levels were higher; this indicates a reduced capacity for rutin metabolism and DHPAA production in the PCOS group (Figure S7E,F). These results suggest that gut microbiota is necessary for rendering the protective effects of DHPAA against PCOS by degrading rutin.

Based on these findings, we further investigated whether the protective effects of DHPAA would be abolished in the absence of gut microbiota. We conducted a mice experiment with a similar design, replacing rutin with DHPAA (Figure S7G,H). We applied DHPAA to PCOS‐like mice with and without antibiotic treatment. The protective effects of DHPAA were observed in PCOS‐like mice in both groups. These effects included a reduction in the number of cystic follicles and testosterone concentration, an increase in the number of corpora lutea, recovery of the estrous cycle, and lower AMH expression levels in ovarian tissue (Figure 4G–L). These findings demonstrate that DHPAA is the end‐product of gut microbiota degrading rutin and exhibits protective effects against PCOS. Taken together, these findings demonstrate that DHPAA, as the end‐product of gut microbiota degrading rutin, exhibits protective effects against PCOS.

### DHPAA mediates the protective effects of *Streptococcus thermophilus* against PCOS

**FIGURE 5:**
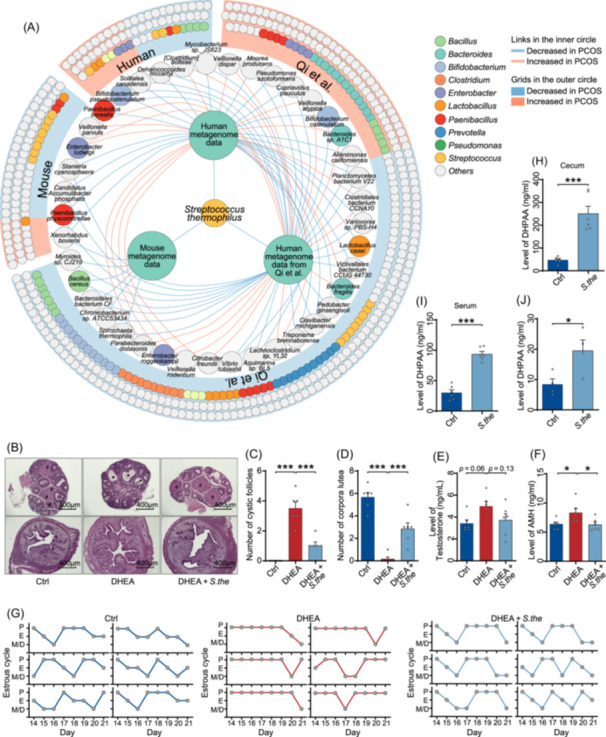
*Streptococcus thermophilus* (*S. thermophilus*) produces 3,4‐dihydroxyphenylacetic acid (DHPAA) and ameliorates polycystic ovary syndrome (PCOS) phenotypes in mice. (A) Differentially abundant bacterial species identified in a discovery human cohort (Human), mice models (Mouse), and an independent validation human cohort (Qi et al.). Each node represents a bacterial species; edges indicate enrichment in PCOS subjects. The outer grid represents all species identified by a consistent analytical pipeline; the inner circle represents the species detected in at least two datasets. (B) Representative H&E‐stained sections of ovaries and uteri from control mice, PCOS‐like mice, and PCOS‐like mice treated with *S. thermophilus* (scale bars, 400 μm). Corpora lutea are indicated by #, and cystic follicle is indicated by *. (C) Quantification of cystic follicles in the ovaries (*n* = 6 mice per group). (D) Quantification of corpora lutea in the ovaries (*n* = 6 mice per group). (E) Levels of testosterone in serum (*n* = 6 mice per group). (F) Levels of AMH in serum (*n* = 6 mice per group). (G) Estrous cycle analysis (*n* = 6 mice per group). (H and I) Concentration of DHPAA in the cecum (H) and serum (I) of mice treated with or without *S. thermophilus* (*n* = 6 mice per group). (J) Concentration of DHPAA in M17 medium with or without *S. thermophilus* (*n* = 4 samples per group). Data are presented as the mean ± SEM. *p < 0.05, **p < 0.01, ***p < 0.001; two‐tailed Student's *t* test; two‐tailed one‐way ANOVA following Dunnett post hoc test.

Next, we investigated changes in gut microbiota of patients with PCOS using metagenomic sequencing on the fecal samples from patients with PCOS and healthy controls. Although no significant difference was found at the community level in our cohort (Figure S8A,B), several bacterial species showed significant differences in relative abundance (Figure S8C). Among there bacteria, *Akkermansia muciniphila*, a high potential novel probiotic [26, 27], and several lactic acid‐producing bacteria were significantly decreased in PCOS group, while taxa belonging to *Fusobacterium* genus, which have been linked to multiple system diseases [28, 29], was significantly increased in PCOS group. Gut microbiota, as one of the most complex communities, is strongly influenced by environmental factors [30, 31, 32]. To obtain more robust bacterial biomarkers and limit the impact of potential variations on gut microbiota, we employed the same analytic pipeline of gut metagenome in another independent cohort from Qi et al. [7], as well as our mice experiment. Integrated analyses identified 36 species that were consistently and significantly different between patients with PCOS and healthy controls in both our human cohort and the independent data set (Figure 5A, and Table S4). Importantly, only one species, *Streptococcus thermophilus* (*S. thermophilus*), showed a consistent and significant reduction in the PCOS group across both human cohorts and the mouse experiment. The decreased relative abundance of *S. thermophilus* was validated in the fecal samples from both human cohort and mice experiment by quantitative PCR (Figure S8D,E). To explore the role of *S. thermophilus* in regulating PCOS, mice were pretreated with *S. thermophilus* by oral gavage for three consecutive days before DHEA injection and once every 2 days afterward (Figure S8F,G). We observed similar protective effects of *S. thermophilus* in the PCOS‐like mice, including a reduction in the number of cystic follicles and the concentration of testosterone, an increase in the number of post‐ovulation corpora lutea, recovery of the estrous cycle, and lower AMH expression level in ovarian tissue (Figure 5B–G). Taken together, we identified a consistent bacterial biomarker, *S. thermophilus*, and demonstrated its protective effects against PCOS‐like symptoms in mice.

Furthermore, although only limited statistically significant associations were observed between the differential bacteria and flavonoid catabolites, there was a positive trend between bacteria decreased in PCOS and flavonoid catabolites, whereas a negative trend was observed for bacteria enriched in PCOS (Figure S8H). This suggests that the alteration of gut bacteria might be related to flavonoid degradation. On the one hand, our results, along with previous studies, demonstrated the connection between gut microbiota and DHPAA, showing that gut bacteria degrade flavonoids into phenolic acids, which enhances their bioavailability and interaction with the host [33]. On the other hand, the capability of *S. thermophilus* to degrade flavonoids has been reported [34], and the necessary enzymes or homologs for this process can be annotated in the genome of *S. thermophilus* according to the UniProt database [35]. Thus, we hypothesized that DHPAA produced by *S. thermophilus* might be protective against PCOS. To test this, we investigated whether *S. thermophilus* could produce DHPAA through in vitro fermentation and in vivo mice experiments. Oral gavage pretreatment of *S. thermophilus* in mice significantly increased DHPAA levels in both cecum and serum samples (Figure 5H,I). *S. thermophilus* produced DHPAA when cultured in M17 medium with mouse chow (Figure 5J). Collectively, our findings suggest the hypothesis that the protective effects of *S. thermophilus* against PCOS may relate to the production of DHPAA.

### β‐galactosidase mediating DHPAA production and protective effects of *S. thermophilus* against PCOS

**FIGURE 6:**
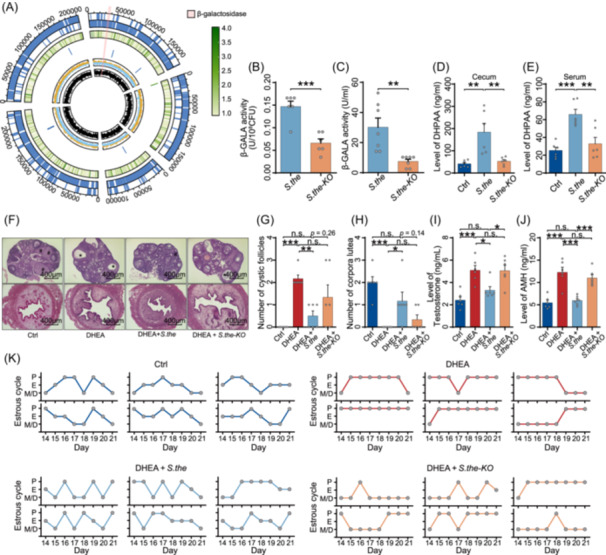
The protective effects of *S. thermophilus* against polycystic ovary syndrome (PCOS) are mediated by β‐galactosidase. (A) Genomic structure and distribution of β‐galactosidase on the chromosome of *S. thermophilus* (pink box). (B) β‐galactosidase activity in bacteria pellets of wild‐type (*S.the*) and β‐galactosidase‐deficient mutant (*S.the*‐KO) strains of *S. thermophilus*. (C) β‐galactosidase activity in culture supernatants of wild‐type and β‐galactosidase‐deficient mutant strains of *S. thermophilus*. (D) DHPAA concentrations in the cecum of control mice, mice treated with *S. thermophilus*, mice treated with β‐galactosidase‐deficient mutant *S. thermophilus* (*n* = 6 mice per group). (E) DHPAA concentrations in the serum of control mice, mice treated with *S. thermophilus*, mice treated with β‐galactosidase‐deficient mutant *S. thermophilus* (*n* = 6 mice per group). (F) Representative H&E‐stained sections of ovaries and uteri from control mice, PCOS‐like mice, PCOS‐like mice pretreated with wild type *S. thermophilus*, and PCOS‐like mice pretreated with β‐GAL‐mutant *S. thermophilus* (scale bars, 400 μm). Corpora lutea are indicated by #, and cystic follicle is indicated by *. (G) Quantification of cystic follicles in the ovaries (*n* = 6 mice per group). (H) Quantification of corpora lutea in the ovaries (*n* = 6 mice per group). (I) Levels of testosterone in serum (*n* = 6 mice per group). (J) Levels of AMH in serum (*n* = 6 mice per group). (K) Estrous cycle analysis (*n* = 6 mice per group). Data are presented as the mean ± SEM. *p < 0.05, **p < 0.01, ***p < 0.001; two‐tailed Student's *t*‐test; two‐tailed one‐way ANOVA following Tukey multiple comparison test.

Given that the protective effects of *S. thermophilus* on PCOS‐like symptoms could be attributed to its capacity to produce DHPAA, we sought to identify the key genes involved in DHPAA production that might contribute to these effects. We sequenced the genome of *S. thermophilus* and identified open reading frames encoding β‐galactosidase, an enzyme implicated in flavonoids degradation [36] (Figure 6A). To investigate whether the protective effects of *S. thermophilus* against PCOS were dependent on β‐galactosidase secretion, a β‐galactosidase‐deficient mutant of the *S. thermophilus* strain was generated using homologous recombination. The reduced expression level of β‐galactosidase was confirmed by PCR (Figure S9A). We verified that the knockout strain of *S. thermophilus* showed decreased β‐galactosidase activity in both culture supernatants and bacterial pellets in vitro (Figure 6B,C). Subsequently, we tested the ability of the *S. thermophilus* knockout strain to produce DHPAA. By culturing both knock‐out and wild‐type strain of *S. thermophilus* with rutin, we found that DHPAA levels were significantly lower in the knock‐out strain compared to the wild‐type strain, while rutin levels were higher. This suggests a reduced capacity for rutin metabolism and DHPAA production in the PCOS group (Figure S9B,C). Additionally, DHPAA levels were remarkably decreased in the cecum and serum samples of mice treated with the knockout strain of *S. thermophilus* compared to the wild‐type strain (Figure 6D,E). Collectively, these results suggest that β‐galactosidase deficiency in *S. thermophilus* leads to decreased production of DHPAA.

Next, we investigated whether this deficiency also compromises the protective effects *S. thermophilus*. PCOS‐like mice were orally administrated either the knock‐out or wild‐type strain of *S. thermophilus* (Figure S9D,E). Quantitative PCR confirmed no significant difference in colonization levels between the two strains (Figure S9F). However, in mice treated with the knockout strain, the ovaries exhibited more cyst‐like follicles and fewer post‐ovulation corpus luteum (Figure 6F,H). Additionally, compared with the wild type group, mice receiving the knockout strain had higher serum testosterone and AMH expression levels, as well as disrupted estrous cycles (Figure 6I–K). Lastly, we assessed fecal β‐galactosidase activity in both PCOS patients and PCOS‐like mice. In both cases, enzyme activity was significantly reduced compared to respective healthy controls (Figure S9G,H). Taken together, these findings indicate that β‐galactosidase is crucial for *S. thermophilus* to produce DHPAA and exert its protective effects against PCOS.

## DISCUSSION

PCOS is recognized as a significant reproductive and metabolic disorder associated with various adverse conditions. Due to the poorly understood mechanisms underlying PCOS, current therapies are largely symptom‐based, highlighting a critical unmet clinical need. A series of studies have suggested that gut microbiota, which is strongly associated with PCOS‐like symptoms in mice, may be a potential cause of the syndrome [7, 9, 37]. Our study contributes to this growing body of knowledge by examining the gut metabolome in patients with PCOS. We found that gut microbiota from PCOS patients show a significant decrease in the ability to produce the gut bacterial‐derived metabolite DHPAA. This metabolite could alleviate PCOS‐like symptoms in mice via inhibiting AMH secretion through the regulation of BMP signaling. Additionally, comprehensive omics analyses and mouse experiments revealed *S. thermophilus* as a potential prophylactic probiotic for PCOS, with its protective effects linked to its ability to produce DHPAA.

Over the past decade, numerous studies have profiled the gut microbiota of patients with PCOS, suggesting its role in the pathogenesis of the syndrome [7, 8, 9]. Emerging research indicates that modifying gut metabolites could be a potential therapeutic target for PCOS. Despite the focus on gut microbiota and PCOS, there is a notable lack of studies on the gut bacterial‐derived metabolites contributing to the condition. While current research primarily targets specific classes of metabolites [38], a comprehensive analysis of the gut metabolomic profiles of PCOS patients is still missing, limiting our understanding of the broader metabolic community. In the present study, we assessed the gut metabolomic profiles of patients with PCOS by an untargeted metabolomics approach. Our findings revealed that the disrupted gut metabolomic profile of patients with PCOS was characterized by reduced correlation and high individual variation in metabolites, rather than differences in the overall composition of metabolite super class compared to healthy controls. There were no statistical differences in the overall metabolite profiles at the superclass/class level between patients and healthy controls. However, the intra‐group variability within the patient group was more pronounced compared to the healthy controls. Specifically, the overall composition reflects inter‐sample variation and was assessed based on metabolite abundance profiles; the co‐occurrence network connectivity captures inter‐metabolite correlation patterns within each group, constructed using a force‐directed layout algorithm based on pairwise associations. These variations highlight the complexity of the gut metabolome in PCOS and underscore the importance of tailoring treatments to the unique metabolic profiles of individuals. Among all the annotated metabolites, several metabolite classes associated with PCOS, including fatty acyls, steroids and steroid derivatives, cinnamic acids and derivatives, flavonoids and polyphenols, and phenols [39, 40, 41, 42, 43, 44, 45]. Additionally, metabolites from the classes of flavonoids, steroids, and pyridines and their derivatives exhibited the largest variations among individuals, with higher variation in the PCOS group compared to the control group. This suggests differences in how the gut microbiota and/or the host processes these metabolites between PCOS patients and healthy controls. Building on previous studies demonstrating the therapeutic potential of modulating bile acid metabolism and agmatine‐related bacteria and metabolites in PCOS [7, 9], our findings further highlight the role of gut microbiota‐derived metabolites as promising targets for PCOS treatment.

As demonstrated in our findings and previous studies, flavonoids are degraded by gut bacteria into phenolic acids, enhancing their bioavailability and thereby exerting various beneficial effects on the host [33, 34]. In our study, consistent with previous reports [46, 47], we found rutin, one of the most common dietary flavonoids found in vegetables and fruits [22], ameliorated PCOS‐like symptoms in mice. Revealing the interaction between gut microbiota and metabolites from an integrated meta‐omics prospective could provide a comprehensive understanding in the crosstalk of gut bacteria and host [48]. Our findings further demonstrated that the beneficial effects of rutin were dependent on its degradation by gut microbiota into downstream derivatives. Taken together, we propose that gut microbiota plays a crucial role in mediating interactions between flavonoids and the host, a factor often neglected in previous studies focusing solely on flavonoids. Our findings provide a deeper understanding of how flavonoids combat diseases and highlight gut microbiota as a critical factor to consider in future research. Microbial‐derived degradation products of flavonoids were reported playing a protective role against diseases, for example, alleviating fatty liver disease, mitigating inflammation, and regulating lipid metabolism [49, 50, 51, 52]. In our study, through metabolomics analysis and in vitro and in vivo validations, we identified DHPAA, a gut bacterial‐derived flavonoid catabolite, as a potential therapeutic candidate for preventing PCOS‐like symptoms in mice. Consistent with a previous cross‐sectional study from Japan [53], we found that the concentration of DHPAA was significantly decreased in patients with PCOS and PCOS‐like mice. Our research further demonstrated that the protective effects of DHPAA were present with or without gut microbiota, indicating that DHPAA is likely the end‐product of gut microbial flavonoid catabolism that ameliorates PCOS. Additionally, both transcriptomic analysis and BMP inhibitor/agonist experiments suggested that DHPAA might reduce AMH secretion by downregulating members of the BMP family. The causal link between DHPAA and the regulation of ovarian gene expression remains to be fully elucidated. DHPAA may influence ovarian function through multiple mechanisms, including direct epigenetic modifications, modulation of transcription factor activity, or indirect effects via systemic metabolic or hormonal changes. Additionally, it remains possible that DHPAA interacts with hormone receptor signaling (e.g., estrogen receptors), which are key regulators of ovarian gene expression and follicle development in PCOS.

Han and colleagues proposed the term “celobiotic” to describe bioactive compounds degraded from the breakdown of dietary components by microbial enzymes, whose biological and pharmacologic activities are independent of further microbial biotransformation [54]. We proposed that DHPAA could be considered a celobiotic, providing valuable insights into the interactions between compounds and microbiota. Compared with bacterial interventions, DHPAA, as a well‐defined small‐molecule metabolite, has the advantage of precise dosing and standardized formulation in pharmacokinetic and toxicological studies, underscoring its translational potential. Additionally, as noted in Han et al. [54], understanding the specific gut microbiota producing a celobiotic could explain interpersonal variations and enable more personalized dietary recommendations with health benefits. Identifying the DHPAA‐producing bacteria could enhance our understanding of its mechanism against PCOS and guide further food and microbiome research. Given the heterogeneity of PCOS phenotypes, future research may explore DHPAA or analogs with enhanced bioavailability, in combination with targeted microbial interventions, to develop more personalized and effective therapeutic strategies.

Recent studies have well‐documented the relationship between gut microbiota and PCOS [8]. Research by Qi et al. and Yun et al. reported that altering gut microbiota or gut‐bacterial derived metabolites could alleviate PCOS‐like symptoms in mice [7, 9], emphasizing the importance of gut microbiota functions in PCOS development. Our study found that the gut microbiota of both patients with PCOS and PCOS‐like mice had a significantly reduced capability to degrade rutin into DHPAA. Given the inevitable variation in gut microbiota influenced by various factors [31, 32], we utilized datasets from our human cohort, mouse experiment, and an independent human cohort from a previous publication. We identified *S. thermophilus* as a consistent bacterial biomarker with protective effects against PCOS. *S. thermophilus*, a thermophilic lactic acid bacterium, is crucial for the food industry [55] and is considered beneficial for alleviating multiple diseases, such as colorectal tumorigenesis, *Clostridium difficile* infection, and depression‐like behaviors [56, 57, 58]. Although limited in number, studies have investigated the mechanisms by which *S. thermophilus* alleviates diseases. For example, *S. thermophilus*, as a representative urease‐producing strain, was reported to reverse stress‐induced depression‐like behaviors [56], and it also inhibits colorectal tumorigenesis by secreting β‐galactosidase [58]. However, in the case of PCOS, despite observations that lactic acid‐producing bacteria can alleviate PCOS [59], research on the underlying mechanisms remains scarce. In this study, based on the evidence that *S. thermophilus* protected against PCOS‐like syndromes in mice, we further discovered that *S. thermophilus* could produce DHPAA through both in vitro and in vivo experiments. Whole‐genome sequencing confirmed that *S. thermophilus* functionally expresses β‐galactosidase, an enzyme involved in flavonoid degradation [36]. Gene knockout of β‐galactosidase in *S. thermophilus* resulted in the loss of its ability to degrade rutin to DHPAA and impaired its protective effects against PCOS. Based on these results, we propose that one mechanism by which *S. thermophilus* protects against PCOS is through the degradation of dietary flavonoids to DHPAA, which subsequently exerts protective effects. Also, in this study, we observed that while *S. thermophilus* showed some protective effects in PCOS‐like mice, its efficacy might be lower than that of direct DHPAA supplementation. Our results indicate that DHPAA acts as the key therapeutic metabolite, leading to a more pronounced effect when supplemented directly. In contrast, *S. thermophilus*, as a DHPAA‐producing bacterium, may be influenced by gut microbiota diversity and environmental factors, leading to variability in its ability to generate sufficient DHPAA for therapeutic effects. Future studies should further investigate how gut microbial composition impacts the efficiency of *S. thermophilus*‐mediated DHPAA production and its role in PCOS treatment.

There are several limitations to this study. Firstly, due to its exploratory and proof‐of‐concept design, the cohort size was relatively small, potentially introducing bias in the gut microbiota results. Future studies with larger populations are needed to provide a more comprehensive interpretation of gut microbiota and evaluate the long‐term safety of DHPAA. We used only rutin as an example of a flavonoid in this study. Since there are various dietary‐originated flavonoids that could be a substrate for DHPAA production by gut microbiota, further validation is required to determine if there is substrate specificity in gut microbial‐derived DHPAA production. Although the concentration of DHPAA in this study was relatively low, it's necessary to test the treatment tolerance to ensure the safety, feasibility, and effectiveness of DHPAA in future preclinical and clinical studies. The DHEA‐induced PCOS‐like mouse model is among the most widely used, as it mimics the key features of PCOS, including hyperandrogenism and disrupts ovulatory function. However, it develops hyperprolactinemia and does not fully replicate the complexity of PCOS in women. Therefore, we employed a LET‐induced model, which more closely resembles the metabolic and hormonal phenotype of PCOS, to further validate the protective effects of DHPAA on PCOS. Future studies will be needed to validate the effects of DHPAA across different animal models and in clinical trials. Estrous cycle stages in this study were not fully synchronized at the time of sacrifice, which may introduce some variability. Future studies will ensure estrous cycle synchronization before tissue collection to minimize this variability. Also, this study cannot distinguish whether ovarian transcriptomic changes induced by DHPAA are direct or indirect effects, requiring further investigation; and the results do not rule out the possibility of other mechanisms of DHPAA alleviating PCOS. For example, compounds with a catechol group have been reported to inhibit 5α‐reductase activity [60], which is upregulated in theca cells in PCOS and related with insulin resistance [61, 62]. Since this study focused on the potential treatment for PCOS but not the etiology of PCOS, future studies are required to explore possible mechanisms by which DHPAA alleviates PCOS. Additionally, the microbial enzymes involved in the metabolism of flavonoids include multiple enzymes, such as the actions of esterases, glycosidases, and reductases [63]. Although we demonstrated that *S. thermophilus* could produce DHPAA via β‐galactosidase, other key enzymes and even unreported enzymes may also play a role in flavonoid degradation and DHPAA generation. In this study, DHPAA was administered concurrently with PCOS induction to evaluate its protective effects; however, future studies are needed to determine whether DHPAA or *S. thermophilus* can serve as independent therapeutic interventions after disease onset. Additionally, the critical role of vaginal microbiota in the reproductive system has been widely reported [64]; further investigations on the potential interactions between microbiota in different body sites and host could provide a deeper understanding in the role of bacteria in PCOS. It should also be mentioned that although the 2003 Rotterdam criteria remain the most commonly applied diagnostic framework for PCOS in both clinical and research settings, recent updates from the 2023 International PCOS Network have raised concerns regarding their potential for overdiagnosis and phenotypic heterogeneity [65]. In particular, the updated consensus highlighted the need to refine the definition of polycystic ovarian morphology and recommended incorporating AMH testing in adult women. Despite these concerns, the Rotterdam criteria are still endorsed by major clinical guidelines and remain widely used in microbiome and metabolomics studies of PCOS. Therefore, we adopted this definition in our study to ensure consistency with previous literature and comparability across cohorts.

## CONCLUSION

In summary, our study provides a comprehensive multi‐omics investigation linking gut microbial metabolism and PCOS. We identified DHPAA as a microbial‐derived celobiotic that ameliorates PCOS‐like phenotypes, in part through suppression of BMP signaling and AMH expression in the ovary. We further demonstrated that *S. thermophilus* contributes to DHPAA production via β‐galactosidase activity, and that loss of this enzyme compromises both metabolite generation and phenotypic rescue. These findings uncover a gut microbiota‐flavonoid‐host signaling axis and propose DHPAA and its microbial producers as promising candidates for future therapeutic development in PCOS management.

## METHODS

### Patients and clinical specimens

We enrolled 30 PCOS patients and 28 healthy volunteers from the First People's Hospital of Foshan in 2022. All participants were females, identified via self‐reported gender. PCOS diagnosis followed the 2003 Rotterdam criteria, requiring at least two of these symptoms: (1) oligo‐ or anovulation, (2) clinical/biochemical hyperandrogenism, and (3) polycystic ovaries, excluding other related disorders like Cushing syndrome, thyroid disease, congenital adrenal hyperplasia. Specifically, the polycystic ovary morphology was defined as the presence of ≥12 follicles measuring 2–9 mm in diameter and/or increased ovarian volume (>10 mL) in at least one ovary, in line with the 2003 Rotterdam criteria. All individuals with PCOS were first‐visit patients and had not received PCOS‐related treatment. Age‐matched healthy controls were selected based on regular menstrual cycles, normal ovarian morphology, and hormone levels. Exclusion criteria included recent use of antibiotics, probiotics, or prebiotics within 3 months before recruitment. Anthropological measures and hormone levels were collected from both patients and controls (Table S1). Written informed consent was obtained from all participants. Serum and fecal samples were collected following informed consent.

### Murine studies

Female C57/BL6 mice, aged 3 weeks and weight 11–13 g, were purchased from SiPeiFu Biotechnology. All mice were housed under specific pathogen‐free conditions with controlled temperature (22 ± 1°C) and humidity (60 ± 10%), on a 12‐h light/dark cycle with free access to food and water. Three or four mice were housed in a standard mouse cage. The mice were randomly divided into experimental groups. To establish a PCOS‐like mouse model, mice received daily subcutaneous injections of DHEA (Macklin; 6 mg per 100 g body weight, dissolved in 0.1 mL sesame oil) for 3 weeks. Additionally, to establish a PCOS‐like mouse model using letrozole as validation, mice received daily oral gavage of letrozole (Aladdin, China; 5 mg per 100 g body weight, dissolved in 1% carboxymethylcellulose solution) for 3 weeks. Control mice were injected with an equivalent volume of sesame oil or oral gavage with carboxymethylcellulose solution over the same period. Body weight was recorded every other day throughout the experiment. Fecal samples were collected at 9:00 am. At the end of the treatment period, mice were euthanized, and tissue samples were collected. Ovaries, uterus, and blood were harvested from each mouse for further analysis.

To deplete the gut microbiota in mice, a broad‐antibiotics regimen was implemented. Mice administered antibiotics (vancomycin, 100 mg/kg; neomycin sulfate, metronidazole, and ampicillin, 200 mg/kg; Macklin) via oral gavage once daily for 3 days before the intervention of DHPAA and DHEA, which was continued every 3 days until the completion of the experiment. To detect whether the protective effects of rutin depend on gut microbiota, mice received an oral gavage of broad antibiotics and 160 mg/kg rutin (Aladdin), which was dissolved in sesame oil (Sigma Aldrige) with 5% DMSO (Sigma Aldrige).

For DHPAA administration, mice were fed with 80 μM or 800 μM DHPAA (Macklin) in drinking water starting 7 days before the DHEA injections and continued throughout the experimental period. For the BMP15 signaling inhibitor experiment, mice were weighed and injected intraperitoneally with 2 mg/kg LDN‐193189 (4‐{6‐[4‐(Piperazin‐1‐yl)phenyl]pyrazolo[1,5‐a]pyrimidin‐3‐yl}quinoline; Selleck) daily which were initiated on Day 10 until the end of the PCOS modeling. For the BMP15 signaling agonist experiment, mice were weighed daily and injected subcutaneously with 1 mg/kg FK506 (Selleck) every other day, which were initiated on Day 1 until the end of the PCOS modeling. For the administration of *S. thermophilus, S. thermophilus* was administered by oral gavage at a dose of 1 × 10^8^ colony‐forming units per 0.2 mL suspended in the PBS, starting 3 days before DHEA‐induced PCOS model and subsequently once every 48 h during the modeling period. The knockout *Streptococcus thermophilus* (KO‐*S. thermophilus*) was administered using the same regimen.

### Fecal incubation experiment

To determine whether the fecal microbiota of PCOS patients had a lower capability in degrading rutin to DHPAA, rutin (at a concentration of 50 μM) was added in PBS and resuspended with fecal samples from control or PCOS groups with a ratio of 1:10 (weight/volume), followed by incubation for 24 h under anaerobic conditions. Finally, DHPAA level was determined immediately after incubation using HPLC analysis.

### Bacterial strains and growth condition

*S. thermophilus* strain was acquired from the BIOBW. For microaerobic cultivation, cells were cultivated statically at 42°C in sterile M17 broth (Macklin), supplemented with 0.001% (w/v) resazurin sodium salt as an anaerobic redox indicator. *S. thermophilus* cultures were collected during mid‐exponential phase (OD₆₀₀ ≈ 0.6–0.8), pelleted by centrifugation (4000 rpm, 10 min, 4°C), and subjected to three washes with ice‐cold sterile PBS. Cell pellets were resuspended in bacterial cryopreservation fluid (75% M17 broth and 25% glycerol) and stored at −80°C until needed. Strain purity was confirmed by 16S rDNA sequencing. Amplification was performed using universal primers 27F (5′‐AGAGTTTGATCMTGGCTCAG‐3′) and 1492R (5′‐TACGGYTACCTTGTTACGACTT‐3′). The resulting sequences were aligned against the EzBioCloud database.

### Gut metabolome and analysis

We collected fecal samples from humans and mice for metabolomic analysis. These samples were processed using a Vanquish UHPLC system equipped with a UPLC BEH Amide column (2.1 mm × 100 mm, 1.7 μm), coupled to a Q Exactive HFX Orbitrap mass spectrometer (Thermo Fisher Scientific). The mobile phase consisted of 25 mM ammonium acetate and 25 mM ammonia hydroxide in water (pH 9.75), paired with acetonitrile. We maintained the auto‐sampler temperature at 4°C, with an injection volume set at 3 μL. The Q Exactive HFX mass spectrometer was selected for its capability to acquire MS/MS spectra through information‐dependent acquisition (IDA) mode, managed by Xcalibur software (Thermo). This software continuously evaluates the full‐scan MS spectrum. Electrospray ionization (ESI) source settings included a sheath gas flow rate of 30 Arb, an auxiliary gas flow rate of 25 Arb, a capillary temperature of 350°C, full MS resolution of 60,000, MS/MS resolution of 7500, collision energy of 10/30/60 in NCE mode, and spray voltages of 3.6 kV (positive) and −3.2 kV (negative). Data management involved rigorous procedures to ensure quality and reliability of the data. These procedures included: (1) Filtering of single peaks to remove noise based on the relative standard deviation (RSD or CV), retaining peaks where no single group had more than 50% null values; (2) Imputing missing values using the minimum half method; (3) Normalizing data against internal standards. For metabolite identification, we utilized a combination of proprietary databases from Magigene and public databases, including the Human Metabolome Database (https://hmdb.ca/). We used the hierarchical classification from HMDB to classify identified metabolites to class and super class. Principal coordinate analysis was performed using the R package ape (v5.7‐1) and vegan (v2.6‐4). Two‐tailed Wilcoxon rank‐sum test and orthogonal partial least squares discriminant analysis (OPLS‐DA) were applied for identification of significantly differential metabolites between groups (OPLS‐DA VIP score > 1, two‐tailed Wilcoxon rank‐sum test *p*‐value < 0.05). To discern variations in community structures, co‐occurrence networks were constructed using ggClusterNet (v0.1.0) [66] with parameters “*N* = 0, *r* = 0.8, *p* = 0.05, method = pearson.” We generated the co‐occurrence networks of the gut metabolites from patients with PCOS and healthy controls and various network topological indices were also calculated using ggClusterNet. The analytic strategy that predicted the function of DHPAA and its potential association with multiple diseases was using an established protocol described in our previous study [14, 67]. In this study, we used the co‐abundance metabolites analytic branch, and the structural similar metabolites analysis branch provided by MeDAM. First, it investigates the biological functions of proteins and genes that interact with the target proteins of the metabolites structurally similar to the queried metabolite, broadening the scope of potential biological interactions. Secondly, it considers metabolites co‐abundant with the queried metabolite to uncover additional potential interacting proteins and genes, thus revealing possible interdependencies. By employing the co‐abundance metabolites analytic branch and the structural similar metabolites analysis branch, we predicted the diseases and pathways that were relevant to DHPAA. The coefficient of variation of each metabolite, defined as the ratio of the standard deviation to the mean, was calculated. We used R package “cvequality” (v0.2.0) to test the differences between coefficients of variation of metabolites using function “mslr_test2” with default parameters (Version 0.2.0; Marwick and Krishnamoorthy 2019) for the MSLRT analysis [68, 69].

### Transcriptome resequencing and analysis

Total RNA was extracted from mouse ovarian tissue using TRIzol reagent. RNA concentration and purity were assessed using a NanoDrop Spectrophotometer (Thermo Scientific). High‐quality RNA samples were then forwarded to Novogene Co., Ltd. for cDNA library construction and subsequent sequencing using the Illumina platform. Quality control of the raw sequencing reads was performed using FastQC (v0.11.9). Adapter trimming and removal of low‐quality bases were carried out using Trimmomatic (0.38). Clean reads were then quantified using Salmon (v1.10.1) against the mouse reference genome GRCm39 (v104). The differential genes were identified using tidybulk (v1.12.0) [70] and DESeq. 2 (v1.40.2) [71] packages with *p*‐value less than 0.01 and fold change greater than 2. To identify biological process involved by the differential genes, the gene ontology enrichment analysis was performed using clusterProfiler (v4.8.3) [72] with org.Mm.eg.db annotation database (v3.19.1). In addition, to further confirm the biological pathway involved by the differential genes, we also performed the Wiki pathway enrichment analysis using clusterProfiler with the Wiki pathway database [73] of mouse and the manually curated database about the biological pathway of *Amh* and *Amhr* gene regulation according to the article [16]. The results were visualized using enrichplot (v1.20.1) [72], ggplot2 (v3.4.4) [74], and ggtree (v3.8.2) [75] packages. A volcano plot of DEGs was generated using the following R packages: ggthemes (v5.1.0) and ggpub (v0.6.0).

### Shotgun metagenome sequencing and analysis

Human and mice fecal samples were sequenced using 150 bp paired end reads on the Illumina NovaSeq. 6000 platform with a target depth of 20 million reads per sample. The metagenomic data from Qi et al.'s work [7] was downloaded from Sequence Read Archive database (https://trace.ncbi.nlm.nih.gov/Traces/home/) via accession number PRJNA530971. All three datasets were analyzed using the same pipeline. Quality control of the reads was performed using fastp (v0.20.1) [76] with the parameter “‐p ‐q 15” to filter adapter contamination and low‐quality reads from the raw sequencing reads and followed by Bowtie2 (v2.4.1) [77] to remove host sequences based on the human genome reference (GRCh38 v100) or the mouse reference genome (GRCm39 v104). The remaining reads were subjected to microbial taxon and gene identification using Kraken2 (v2.1.2) [78] and HuMAnN3 (v3.0.0. alpha.3) [79]. Alpha and beta diversities were calculated using MicrobiotaProcess (v1.13.2.994) [80]. The differential bacteria species were identified using DESeq. 2 (v1.40.2) [71] packages with Benjamini–Hochberg method adjusted *p*‐value less than 0.2. Considering the high dimensionality, sparsity, and interindividual variability inherent to metagenomic data, we adapted this more lenient threshold to improve sensitivity in detecting potentially relevant taxa without excessively inflating type I error. Additionally, downstream validation across independent cohorts, in vivo experiments, and functional analyses were performed to enhance the robustness of taxonomic associations. The correlations between flavonoids derivatives and gut bacterial species were performed based on Spearman's rank correlation coefficient (*ρ*). A volcano plot of differential bacteria species was generated using the R package ggplot2 (v3.4.4) and ggrepel (v0.9.3).

### Construction of the β‐galactosidase knockout *S. thermophilus* strain

A homologous recombination technique was applied to construct the β‐galactosidase (*β‐gal*) knockout *S. thermophilus* strain. Briefly, the erythromycin resistance (*Erm*) gene was synthesized and cloned, along with 500‐bp upstream and 400‐bp downstream flanking regions of the *β‐gal* gene, into the EcoRV site of plasmid pUC57 (Geneyuan Co. Ltd.) to generate the homologous recombination plasmid. For competent cell preparation, *S. thermophilus* was cultured in M17 medium overnight to reach an OD600 absorbance of 0.8. Cells were harvested by centrifugation (4000 rpm, 5 min), resuspended in 10 mL of pre‐warmed M17 broth containing 0.5% glycine, and incubated microaerobically at 42°C for 2 h. After chilling on ice for 15 min, cells were harvested (4000 rpm, 5 min) to obtain competent cells. The recombinant plasmid was electroporated (1.8 kV, 2.5 ms) into glycine‐treated competent cells using electroporation buffer (0.5 mol/L sucrose, 1 mmol/L ammonium citrate, pH 6.0). The electroporated *S. thermophilus* were spread onto M17 agar containing erythromycin (7.5 μg/mL) for isolation. The colonies were identified by colony PCR and agarose gel electrophoresis. Primes of the *Erm* were F‐5′‐GTAAAACGACGGCCAGTG‐3′ and R‐5′‐GGAAACAGCTATGACCATG‐3′.

### Quantification by real‐time qPCR

Real‐time qPCR analysis was performed using the SYBR Green master mix use the Applied Biosystems 7500 real‐time PCR system according to the manufacturer's instructions. The amplification thermal cycling conditions were as follows: initial denaturation at 95°C for 10 min; 40 cycles of denaturation at 95°C for 15 s, annealing at 60°C for 15 s, and extension at 72°C for 45 s. The qPCR primers used in this study can be found in Table S5. Relative gene expression levels were quantified and normalized using the 2^−ΔΔCt^ method.

### Ovarian histological analysis

Ovaries were collected from mice, immersion‐fixed in 4% polyformaldehyde, and placed in 70% ethanol, dehydrated, and embedded in paraffin. Sagittal sections (3 μm thickness) were cut along the cephalocaudal axis and stained with hematoxylin‐eosin. Quantification of corpora lutea and cystic follicles was performed by a pathologist under blinded conditions.

### Vaginal lavages and estrous cycle determination

For two consecutive estrous cycles, vaginal samples were taken daily at 09:00 from the 14th to the 21st day (or 8th to the 21st day in the letrozole induced PCOS‐like mice experiment) after the first day of the treatment. The stage of the estrous cycle was determined by microscopic analysis of the predominant cell tyle in vaginal lavages. Smears were collected using 10 μL of 0.9% sterile saline and transferred to glass slides to air dry. Dry smears were stained with 1% crystal violet before examination under a light microscope. Estrous cycle stage was determined based on the presence or absence of leukocytes, cornified epithelial cells, and nucleated epithelial cells. Proestrus was characterized by the presence of mainly nucleated and some cornified epithelial cells; the estrus stage exhibited cornified epithelial cells; the metestrus stage was marked by the presence of both cornified epithelial cells and leukocytes; and diestrus stage was typified by the presence of predominantly leukocytes. The estrous cycles were assessed by one author and validated by another, both of whom were blinded to the group assignments.

### Biochemical and ELISA assays

The blood samples were centrifuged at 3000 rpm for 15 min at 4°C and stored at −80°C for subsequent serum determinations. The levels of testosterone (T) and anti‐Müllerian hormone were determined by enzyme‐linked immunosorbent assay kits for mice (FineTest). The activities of β‐galactosidase were detected using the commercial kits from Solarbio.

### Liquid chromatography‐tandem mass spectrometry

Serum and fecal samples from humans, as well as cecum and serum samples from mice, were precipitated using mass spectrometry‐grade methanol. After precipitation, the concentration of DHPAA in the supernatant was measured via ultra‐performance liquid chromatography (Waters). The system utilized a 2.1 × 5.0 mm, 1.8 μm Waters ACQUITY column operated at 35°C. The mobile phases were 0.1% formic acid (Mobile Phase A) an acetonitrile (Mobile Phase B). The parent ion had an *m*/*z* value of 167, and the daughter ion had an *m*/*z* value of 123. All fecal and serum samples were aliquoted and immediately stored at −80°C upon collection to prevent metabolite degradation. During extraction, samples were processed on ice and protected from light to minimize oxidation.

### Quantification and statistical analysis

Statistical analyses were conducted using GraphPad Prism (v10.2.0.392) and R (v4.3.1). For comparisons between two groups, we used either two‐tailed Wilcoxon rank‐sum test or two‐tailed Student's *t*‐test, depending on the data type. Multi‐group comparisons were analyzed using two‐tailed one‐way ANOVA followed with Dunnett's test for multiple group adjustment. Correlation analyses employed Spearman's rank correlation coefficient (*ρ*). The Benjamini and Hochberg method was applied to adjust for multiple hypothesis testing [81]. Results were considered statistically significant at *p* < 0.05, with significance levels indicated as *p < 0.05, **p < 0.01, and ***p < 0.001.

## AUTHOR CONTRIBUTIONS

**Pan Li**: Conceptualization; investigation; Writing—original draft; Writing—review and editing; visualization; methodology; formal analysis; data curation. **Li Xie**: Investigation; Writing—review and editing; methodology; validation; formal analysis; data curation. **Huimin Zheng**: Conceptualization; investigation; funding acquisition; Writing—original draft; visualization; methodology; software; formal analysis; data curation; supervision; resources; Writing—review and editing. **Yinglin Feng**: Investigation; Writing—review and editing; methodology; validation; formal analysis; project administration; data curation. **Feihong Mai**: Investigation; methodology; validation; Writing—review and editing; data curation. **Wenli Tang**: Investigation; Writing—review and editing; visualization; validation; methodology; software; formal analysis; data curation. **Jiajia Wang**: Investigation; Writing—review and editing; validation; methodology; data curation. **Zixin Lan**: Validation; investigation; Writing—review and editing; methodology; data curation. **Shuaijun Lv**: Investigation; validation. **Thisun Jayawardana**: Writing—review and editing; Writing—original draft. **Sabrina Koentgen**: Writing—review and editing; Writing—original draft. **Shuangbin Xu**: Visualization. **Zhengwei Wan**: Writing—review and editing. **Yunjie Chen**: Writing—review and editing. **Haiyan Xu**: Writing—review and editing. **Sj Shen**: Writing—review and editing; Writing—original draft. **Fan Zhang**: Writing—review and editing. **Yuanhao Yang**: Writing—review and editing. **Georgina Hold**: Writing—review and editing. **Fangjie He**: Writing—review and editing; supervision; resources. **Emad M. El‐Omar**: Writing—review and editing; Writing—original draft; supervision; resources; project administration. **Guangchuang Yu**: Supervision. **Xia Chen**: Conceptualization; funding acquisition; Writing—review and editing; data curation; validation; methodology; project administration; supervision; resources.

## CONFLICT OF INTEREST STATEMENT

The authors declare no conflicts of interest.

## ETHICS STATEMENT

The study was ethically approved by the committee of the First People's Hospital of Foshan (Approval No. FSYYY‐EC‐SOP‐008‐02.0‐A09). All experimental protocols involving animals were performed under the National Institutes of Health guidelines and were approved by the local Animal Care and Use Committee of the First People's Hospital of Foshan (Approval No. 20220314‐sr‐mouse‐2).

## Supporting information


**Figure S1:** Metabolite composition of patients and mice with PCOS.
**Figure S2:** DHPAA relates to reproductive system diseases and is decreased in PCOS.
**Figure S3:** DHPAA alleviates PCOS‐like symptoms in LET‐induce PCOS‐like mice.
**Figure S4:** Protective effects of DHPAA against PCOS links to inhibiting BMP signaling.
**Figure S5:** The use of BMP signaling agonist abolishes protective effects of DHPAA against PCOS.
**Figure S6:** The use of BMP signaling agonist abolishes protective effects of DHPAA against PCOS.
**Figure S7:** DHPAA Production depends on the existence of gut microbiota.
**Figure S8:** *Streptococcus thermophilus* is identified as a biomarker for PCOS and links to flavonoid degradation.
**Figure S9:** β‐galactosidase activity is reduced in PCOS.

**Table S1:** Summary descriptives table by groups.
**Table S2:** Network properties of co‐occurrence network analyses.
**Table S3:** The coefficients of variation of metabolites and Modified Signed‐likelihood Ratio test between groups.
**Table S4:** The enriched/decreased bacteria between groups in the human cohort, mice experiment, and Qi's dataset.
**Table S5:** Primers used in qPCR analysis.

## Data Availability

The data that support the findings of this study are openly available in the Chinese National Gene Bank Nucleotide Sequence Archive at https://db.cngb.org/cnsa/, reference numbers CNP0006050 and CNP0006039. The metagenome data have been deposited in the Chinese National Gene Bank Nucleotide Sequence Archive under accession numbers CNP0006050 and CNP0006039 (https://db.cngb.org/search/project/CNP0006050 and https://db.cngb.org/search/project/CNP0006039). The data and scripts used are saved in GitHub (https://github.com/WENLITANG/PCOS_SupplementaryFile). Supplementary materials (figures, tables, graphical abstract, slides, videos, Chinese translated version, and update materials) may be found in the online DOI or iMeta Science http://www.imeta.science/.

## References

[1] Azziz, Ricardo , Enrico Carmina , ZiJiang Chen , Andrea Dunaif , Joop S. E. Laven , Richard S. Legro , Daria Lizneva , et al. 2016. “Polycystic Ovary Syndrome.” Nature Reviews Disease Primers 2: 16057. 10.1038/nrdp.2016.57 27510637

[2] Stener‐Victorin, Elisabet , Helena Teede , Robert J. Norman , Richard Legro , Mark O. Goodarzi , Anuja Dokras , Joop Laven , Kathleen Hoeger , and Terhi T. Piltonen . 2024. “Polycystic Ovary Syndrome.” Nature Reviews Disease Primers 10: 27. 10.1038/s41572-024-00511-3 38637590

[3] Joham, Anju E. , Robert J. Norman , Elisabet Stener‐Victorin , Richard S. Legro , Stephen Franks , Lisa J. Moran , Jacqueline Boyle , and Helena J. Teede . 2022. “Polycystic Ovary Syndrome.” Lancet Diabetes & Endocrinology 10: 668–680. 10.1016/s2213-8587(22)00163-2 35934017

[4] Wan, Zhengwei , Jianhui Zhao , Yongju Ye , Zhaochen Sun , Kangning Li , Yan Chen , and Yuan Fang , et al. 2024. “Risk and Incidence of Cardiovascular Disease Associated With Polycystic Ovary Syndrome.” European Journal of Preventive Cardiology 31: 1560–1570. 10.1093/eurjpc/zwae066 38373259

[5] Escobar‐Morreale, Héctor F . 2018. “Polycystic Ovary Syndrome: Definition, Aetiology, Diagnosis and Treatment.” Nature Reviews Endocrinology 14: 270–284. 10.1038/nrendo.2018.24 29569621

[6] Torres, Pedro J. , Martyna Siakowska , Beata Banaszewska , Leszek Pawelczyk , Antoni J. Duleba , Scott T. Kelley , and Varykina G. Thackray . 2018. “Gut Microbial Diversity in Women With Polycystic Ovary Syndrome Correlates With Hyperandrogenism.” Journal of Clinical Endocrinology & Metabolism 103: 1502–1511. 10.1210/jc.2017-02153 29370410 PMC6276580

[7] Qi, Xinyu , Chuyu Yun , Lulu Sun , Jialin Xia , Qing Wu , Ying Wang , Lina Wang , et al. 2019. “Gut Microbiota–Bile Acid–Interleukin‐22 Axis Orchestrates Polycystic Ovary Syndrome.” Nature Medicine 25: 1225–1233. 10.1038/s41591-019-0509-0 PMC7376369 31332392

[8] Li, Pan , Ping Shuai , Sj Shen , Huimin Zheng , Ping Sun , Renfang Zhang , Shanwei Lan , et al. 2023. “Perturbations in Gut Microbiota Composition in Patients With Polycystic Ovary Syndrome: A Systematic Review and Meta‐Analysis.” BMC Medicine 21: 302. 10.1186/s12916-023-02975-8 37559119 PMC10413517

[9] Yun, Chuyu , Sen Yan , Baoying Liao , Yong Ding , Xinyu Qi , Min Zhao , Kai Wang , et al. 2024. “The Microbial Metabolite Agmatine Acts as an FXR Agonist to Promote Polycystic Ovary Syndrome in Female Mice.” Nature Metabolism 6: 947–962. 10.1038/s42255-024-01041-8 38769396

[10] Shoaei, Tanaz , Motahar Heidari‐Beni , HatavGhasemi Tehrani , Awat feizi , Ahmad Esmaillzadeh , and Gholamreza Askari . 2015. “Effects of Probiotic Supplementation on Pancreatic β‐Cell Function and C‐Reactive Protein in Women With Polycystic Ovary Syndrome: A Randomized Double‐Blind Placebo‐Controlled Clinical Trial.” International Journal of Preventive Medicine 6: 27. 10.4103/2008-7802.153866 25949777 PMC4387688

[11] Jamilian, Mehri , Shirin Mansury , Fereshteh Bahmani , Zahra Heidar , Elaheh Amirani , and Zatollah Asemi . 2018. “The Effects of Probiotic and Selenium Co‐Supplementation on Parameters of Mental Health, Hormonal Profiles, and Biomarkers of Inflammation and Oxidative Stress in Women With Polycystic Ovary Syndrome.” Journal of Ovarian Research 11: 80. 10.1186/s13048-018-0457-1 30217229 PMC6137747

[12] Chen, Mingyue , Chengyong He , Kongyang Zhu , Zihan Chen , Zixiao Meng , Xiaoming Jiang , Jiali Cai , Chunyan Yang , and Zhenghong Zuo . 2022. “Resveratrol Ameliorates Polycystic Ovary Syndrome via Transzonal Projections Within Oocyte‐Granulosa Cell Communication.” Theranostics 12: 782–795. 10.7150/thno.67167 34976213 PMC8692920

[13] Feng, Xinchi , Yang Li , Mahmood Brobbey Oppong , and Feng Qiu . 2018. “Insights Into the Intestinal Bacterial Metabolism of Flavonoids and the Bioactivities of Their Microbe‐Derived Ring Cleavage Metabolites.” Drug Metabolism Reviews 50: 343–356. 10.1080/03602532.2018.1485691 30010437

[14] Zheng, Huimin , Feihong Mai , Siyou Zhang , Zixin Lan , Zhang Wang , Shanwei Lan , Renfang Zhang , et al. 2024. “In Silico Method to Maximise the Biological Potential of Understudied Metabolomic Biomarkers: A Study in Pre‐Eclampsia.” Gut 73: 383–385. 10.1136/gutjnl-2022-329312 36725314

[15] Liu, Yang , Jing‐jing Jiang , Shao‐yue Du , Liang‐shan Mu , Jian‐jun Fan , Jun‐chi Hu , Yao Ye , et al. 2024. “Artemisinins Ameliorate Polycystic Ovarian Syndrome by Mediating LONP1‐CYP11A1 Interaction.” Science 384: eadk5382. 10.1126/science.adk5382 38870290

[16] di Clemente, Nathalie , Chrystèle Racine , Alice Pierre , and Joëlle Taieb . 2021. “Anti‐Müllerian Hormone in Female Reproduction.” Endocrine Reviews 42: 753–782. 10.1210/endrev/bnab012 33851994

[17] Roy, Sambit , Divya Gandra , Christina Seger , Anindita Biswas , Vitaly A. Kushnir , Norbert Gleicher , T. Rajendra Kumar , and Aritro Sen . 2018. “Oocyte‐Derived Factors (GDF9 and BMP15) and FSH Regulate AMH Expression via Modulation of H3K27AC in Granulosa Cells.” Endocrinology 159: 3433–3445. 10.1210/en.2018-00609 30060157 PMC6112599

[18] Zhao, Zhongquan , Fangyue Guo , Xiaowei Sun , Qijie He , Zinuo Dai , Xiaochuan Chen , Yongju Zhao , and Jian Wang . 2018. “BMP15 Regulates AMH Expression via the p38 MAPK Pathway in Granulosa Cells From Goat.” Theriogenology 118: 72–79. 10.1016/j.theriogenology.2018.05.032 29885643

[19] Tata, Brooke , Nour El Houda Mimouni , Anne‐Laure Barbotin , Samuel A. Malone , Anne Loyens , Pascal Pigny , Didier Dewailly , et al. 2018. “Elevated Prenatal Anti‐Müllerian Hormone Reprograms the Fetus and Induces Polycystic Ovary Syndrome in Adulthood.” Nature Medicine 24: 834–846. 10.1038/s41591-018-0035-5 PMC6098696 29760445

[20] Mimouni, Nour El Houda , Isabel Paiva , Anne‐Laure Barbotin , Fatima Ezzahra Timzoura , Damien Plassard , Stephanie Le Gras , Gaetan Ternier , et al. 2021. “Polycystic Ovary Syndrome Is Transmitted via a Transgenerational Epigenetic Process.” Cell Metabolism 33: 513–530.e8. 10.1016/j.cmet.2021.01.004 33539777 PMC7928942

[21] Dias, Maria Celeste , Diana C. G. A. Pinto , and Artur M. S. Silva . 2021. “Plant Flavonoids: Chemical Characteristics and Biological Activity.” Molecules 26: 5377. 10.3390/molecules26175377 34500810 PMC8434187

[22] Farha, Arakkaveettil Kabeer , Ren‐You Gan , Hua‐Bin Li , Ding‐Tao Wu , Atanas G. Atanasov , Khalid Gul , Jia‐Rong Zhang , Qiong‐Qiong Yang , and Harold Corke . 2020. “The Anticancer Potential of the Dietary Polyphenol Rutin: Current Status, Challenges, and Perspectives.” Critical Reviews in Food Science and Nutrition 62: 832–859. 10.1080/10408398.2020.1829541 33054344

[23] Catalán, Mabel , Jorge Ferreira , and Catalina Carrasco‐Pozo . 2020. “The Microbiota‐Derived Metabolite of Quercetin, 3,4‐Dihydroxyphenylacetic Acid Prevents Malignant Transformation and Mitochondrial Dysfunction Induced by Hemin in Colon Cancer and Normal Colon Epithelia Cell Lines.” Molecules 25: 4138. 10.3390/molecules25184138 32927689 PMC7571211

[24] Murota, Kaeko , Yoshimasa Nakamura , and Mariko Uehara . 2018. “Flavonoid Metabolism: The Interaction of Metabolites and Gut Microbiota.” Bioscience, Biotechnology, and Biochemistry 82: 600–610. 10.1080/09168451.2018.1444467 29504827

[25] Han, Qixin , Juan Wang , Weiping Li , Zi‐Jiang Chen , and Yanzhi Du . 2021. “Androgen‐Induced Gut Dysbiosis Disrupts Glucolipid Metabolism and Endocrinal Functions in Polycystic Ovary Syndrome.” Microbiome 9: 101. 10.1186/s40168-021-01046-5 33957990 PMC8103748

[26] Xie, Shihao , Jiaxin Li , Fengyuan Lyu , Qingming Xiong , Peng Gu , Yuqi Chen , Meiling Chen , et al. 2024. “Novel Tripeptide RKH Derived From *Akkermansia muciniphila* Protects Against Lethal Sepsis.” Gut 73: 78–91. 10.1136/gutjnl-2023-329996 37553229

[27] Cani, Patrice D. , Clara Depommier , Muriel Derrien , Amandine Everard , and Willem M. de Vos . 2022. “ *Akkermansia muciniphila*: Paradigm for Next‐Generation Beneficial Microorganisms.” Nature Reviews Gastroenterology & Hepatology 19: 625–637. 10.1038/s41575-022-00631-9 35641786

[28] Manson McGuire, Abigail , Kyla Cochrane , Allison D. Griggs , Brian J. Haas , Thomas Abeel , Qiandong Zeng , Justin B. Nice , et al. 2014. “Evolution of Invasion in a Diverse Set of *Fusobacterium* Species.” mBio 5: e01864. 10.1128/mBio.01864-14 25370491 PMC4222103

[29] He, Yan , Prabhakar Mujagond , Wenli Tang , Wei Wu , Huimin Zheng , Xia Chen , Muxuan Chen , et al. 2021. “Non‐Nucleatum *Fusobacterium* Species Are Dominant in the Southern Chinese Population With Distinctive Correlations to Host Diseases Compared With F. Nucleatum.” Gut 70: 810–812. 10.1136/gutjnl-2020-322090 32690601

[30] Gacesa, R. , A. Kurilshikov , A. Vich Vila , T. Sinha , M. A. Y. Klaassen , L. A. Bolte , S. Andreu‐Sánchez , et al. 2022. “Environmental Factors Shaping the Gut Microbiome in a Dutch Population.” Nature 604: 732–739. 10.1038/s41586-022-04567-7 35418674

[31] Gaulke, Christopher A. , and Thomas J. Sharpton . 2018. “The Influence of Ethnicity and Geography on Human Gut Microbiome Composition.” Nature Medicine 24: 1495–1496. 10.1038/s41591-018-0210-8 30275567

[32] He, Yan , Wei Wu , Hui‐Min Zheng , Pan Li , Daniel McDonald , Hua‐Fang Sheng , Mu‐Xuan Chen , et al. 2018. “Regional Variation Limits Applications of Healthy Gut Microbiome Reference Ranges and Disease Models.” Nature Medicine 24: 1532–1535. 10.1038/s41591-018-0164-x 30150716

[33] Thilakarathna, Surangi , and H. Rupasinghe . 2013. “Flavonoid Bioavailability and Attempts for Bioavailability Enhancement.” Nutrients 5: 3367–3387. 10.3390/nu5093367 23989753 PMC3798909

[34] He, Zhishu , Hao Zhang , Tao Wang , Ren Wang , and Xiaohu Luo . 2022. “Effects of Five Different Lactic Acid Bacteria on Bioactive Components and Volatile Compounds of Oat.” Foods 11: 3230. 10.3390/foods11203230 37430979 PMC9602019

[35] Bateman, Alex , Maria‐Jesus Martin , Sandra Orchard , Michele Magrane , Shadab Ahmad , Emanuele Alpi , Emily H. Bowler‐Barnett , et al. 2023. “UniProt: The Universal Protein Knowledgebase in 2023.” Nucleic Acids Research 51: D523–D531. 10.1093/nar/gkac1052 36408920 PMC9825514

[36] Ozdal, Tugba , David A. Sela , Jianbo Xiao , Dilek Boyacioglu , Fang Chen , and Esra Capanoglu . 2016. “The Reciprocal Interactions Between Polyphenols and Gut Microbiota and Effects on Bioaccessibility.” Nutrients 8: 78. 10.3390/nu8020078 26861391 PMC4772042

[37] Wu, Jiayu , Kai Wang , Xinyu Qi , Shuang Zhou , Shuyun Zhao , Meisong Lu , Qixing Nie , et al. 2025. “The Intestinal Fungus Aspergillus Tubingensis Promotes Polycystic Ovary Syndrome Through a Secondary Metabolite.” Cell Host & Microbe 33: 119–136.e111. 10.1016/j.chom.2024.12.006 39788092

[38] Rajska, Anna , Magdalena Buszewska‐Forajta , Dominik Rachoń , and Michał Jan Markuszewski . 2020. “Metabolomic Insight Into Polycystic Ovary Syndrome—An Overview.” International Journal of Molecular Sciences 21: 4853. 10.3390/ijms21144853 32659951 PMC7402307

[39] Cree‐Green, Melanie , Anne‐Marie Carreau , Haseeb Rahat , Yesenia Garcia‐Reyes , Bryan C. Bergman , Laura Pyle , and Kristen J. Nadeau . 2019. “Amino Acid and Fatty Acid Metabolomic Profile During Fasting and Hyperinsulinemia in Girls With Polycystic Ovarian Syndrome.” American Journal of Physiology‐Endocrinology and Metabolism 316: E707–E718. 10.1152/ajpendo.00532.2018 30753112 PMC6580169

[40] Maleki, Vahid , Amir Hossein Faghfouri , Fatemeh Pourteymour Fard Tabrizi , Jalal Moludi , Sevda Saleh‐Ghadimi , Hamed Jafari‐Vayghan , and Shaimaa A. Qaisar . 2021. “Mechanistic and Therapeutic Insight Into the Effects of Cinnamon in Polycystic Ovary Syndrome: A Systematic Review.” Journal of Ovarian Research 14: 130. 10.1186/s13048-021-00870-5 34627352 PMC8502340

[41] Hussain, Liaqat , Noor Aamir , Musaddique Hussain , Muhammad Asif , Zunera Chauhdary , Faiza Manzoor , Rida Siddique , Muhammad Riaz , and Alamgeer Yuchi . 2022. “Therapeutic Investigation of Standardized Aqueous Methanolic Extract of Bitter Melon (*Momordica charantia L.*) for Its Potential Against Polycystic Ovarian Syndrome in Experimental Animals' Model: In Vitro and In Vivo Studies.” Evidence‐Based Complementary and Alternative Medicine 2022: 1–14. 10.1155/2022/5143653 PMC9536891 36212951

[42] Zhang, Jiacheng , Haolin Zhang , Xiyan Xin , Yutian Zhu , Yang Ye , and Dong Li . 2022. “Efficacy of Flavonoids on Animal Models of Polycystic Ovary Syndrome: A Systematic Review and Meta‐Analysis.” Nutrients 14: 4128. 10.3390/nu14194128 36235780 PMC9571610

[43] Mihanfar, Aynaz , Mohammad Nouri , Leila Roshangar , and Mohammad Hassan Khadem‐Ansari . 2021. “Polyphenols: Natural Compounds With Promising Potential in Treating Polycystic Ovary Syndrome.” Reproductive Biology 21: 100500. 10.1016/j.repbio.2021.100500 33878526

[44] Delgado, Amélia Martins , Manel Issaoui , and Nadia Chammem . 2019. “Analysis of Main and Healthy Phenolic Compounds in Foods.” Journal of AOAC International 102: 1356–1364. 10.5740/jaoacint.19-0128 31200788

[45] Palioura, Eleni , and Evanthia Diamanti‐Kandarakis . 2016. “Polycystic Ovary Syndrome (PCOS) and Endocrine Disrupting Chemicals (EDCs).” Reviews in Endocrine and Metabolic Disorders 16: 365–371. 10.1007/s11154-016-9326-7 26825073

[46] Jahan, Sarwat , Faryal Munir , Suhail Razak , Anam Mehboob , Qurat Ul Ain , Hizb Ullah , Tayyaba Afsar , Ghazala Shaheen , and Ali Almajwal . 2016. “Ameliorative Effects of Rutin Against Metabolic, Biochemical and Hormonal Disturbances in Polycystic Ovary Syndrome in Rats.” Journal of Ovarian Research 9: 86. 10.1186/s13048-016-0295-y 27923406 PMC5142269

[47] Hu, Tao , Xiaoxue Yuan , Rongcai Ye , Huiqiao Zhou , Jun Lin , Chuanhai Zhang , Hanlin Zhang , et al. 2017. “Brown Adipose Tissue Activation by Rutin Ameliorates Polycystic Ovary Syndrome in Rat.” Journal of Nutritional Biochemistry 47: 21–28. 10.1016/j.jnutbio.2017.04.012 28501702

[48] Shi, Chun‐Lin , Tong Chen , Canhui Lan , Ren‐You Gan , Jun Yu , Fangqing Zhao , and Yong‐Xin Liu . 2024. “iMetaOmics: Advancing Human and Environmental Health Through Integrated Meta‐Omics.” iMetaOmics 1: 1. 10.1002/imo2.21 PMC12806273 41675548

[49] Ding, Huafang , Jianhui Liu , Zixing Chen , Shouhe Huang , Chi Yan , Erika Kwek , Zouyan He , Hanyue Zhu , and Zhen‐Yu Chen . 2024. “Protocatechuic Acid Alleviates TMAO‐Aggravated Atherosclerosis via Mitigating Inflammation, Regulating Lipid Metabolism, and Reshaping Gut Microbiota.” Food & Function 15: 881–893. 10.1039/d3fo04396g 38165856

[50] An, Sheng , Yi Yao , Junjie Wu , Hongbin Hu , Jie Wu , Maomao Sun , Jiaxin Li , et al. 2024. “Gut‐Derived 4‐Hydroxyphenylacetic Acid Attenuates Sepsis‐Induced Acute Kidney Injury by Upregulating ARC to Inhibit Necroptosis.” Biochimica et Biophysica Acta (BBA) ‐ Molecular Basis of Disease 1870: 166876. 10.1016/j.bbadis.2023.166876 37714058

[51] Gu, Peng , Ruofan Liu , Qin Yang , Li Xie , Rongjuan Wei , Jiaxin Li , Fengyi Mei , et al. 2023. “A Metabolite From Commensal *Candida Albicans* Enhances the Bactericidal Activity of Macrophages and Protects Against Sepsis.” Cellular & Molecular Immunology 20: 1156–1170. 10.1038/s41423-023-01070-5 37553429 PMC10541433

[52] Tan, Jijun , Ruizhi Hu , Jiatai Gong , Chengkun Fang , Yanli Li , Ming Liu , Ziyu He , et al. 2023. “Protection Against Metabolic Associated Fatty Liver Disease by Protocatechuic Acid.” Gut Microbes 15: 2238959. 10.1080/19490976.2023.2238959 37505920 PMC10392757

[53] Yoshino, K. , K. Takahashi , Y. Eda , A. Nishigaki , and M. Kitao . 1993. “Peripheral Catecholamine Metabolites and Menstrual Irregularity in Patients With Polycystic Ovaries.” Int Journal of Fertility and Menopausal Studies 38: 225–228. https://www.ncbi.nlm.nih.gov/pubmed/8401681 8401681

[54] Han, Nathan D. , Jiye Cheng , Omar Delannoy‐Bruno , Daniel Webber , Nicolas Terrapon , Bernard Henrissat , Dmitry A. Rodionov , et al. 2022. “Microbial Liberation of N‐Methylserotonin From Orange Fiber in Gnotobiotic Mice and Humans.” Cell 185: 2495–2509.e11. 10.1016/j.cell.2022.06.004 35764090 PMC9271604

[55] Hols, Pascal , Frédéric Hancy , Laetitia Fontaine , Benoît Grossiord , Deborah Prozzi , Nathalie Leblond‐Bourget , Bernard Decaris , et al. 2005. “New Insights in the Molecular Biology and Physiology of *Streptococcus thermophilus* Revealed by Comparative Genomics.” FEMS Microbiology Reviews 29: 435–463. 10.1016/j.fmrre.2005.04.008 16125007

[56] Wang, Pei , Peng‐Fei Wu , Hua‐Jie Wang , Fang Liao , Fang Wang , and Jian‐Guo Chen . 2023. “Gut Microbiome‐Derived Ammonia Modulates Stress Vulnerability in the Host.” Nature Metabolism 5: 1986–2001. 10.1038/s42255-023-00909-5 37872351

[57] Kolling, Glynis L. , Martin Wu , Cirle A. Warren , Evelyn Durmaz , Todd R. Klaenhammer , and Richard L. Guerrant . 2014. “Lactic Acid Production by *Streptococcus thermophilus* Alters Clostridium Difficile Infection and In Vitro Toxin A Production.” Gut Microbes 3: 523–529. 10.4161/gmic.21757 PMC3495789 22895082

[58] Li, Qing , Wei Hu , Wei‐Xin Liu , Liu‐Yang Zhao , Dan Huang , Xiao‐Dong Liu , Hung Chan , et al. 2021. “ *Streptococcus thermophilus* Inhibits Colorectal Tumorigenesis Through Secreting β‐Galactosidase.” Gastroenterology 160: 1179–1193.e14. 10.1053/j.gastro.2020.09.003 32920015

[59] He, Yufeng , Qianqian Wang , Xiu Li , Gang Wang , Jianxin Zhao , Hao Zhang , and Wei Chen . 2020. “Lactic Acid Bacteria Alleviate Polycystic Ovarian Syndrome by Regulating Sex Hormone Related Gut Microbiota.” Food & Function 11: 5192–5204. 10.1039/c9fo02554e 32441726

[60] Hiipakka, Richard A. , Han‐Zhong Zhang , Wei Dai , Qing Dai , and Shutsung Liao . 2002. “Structure–Activity Relationships for Inhibition of Human 5α‐Reductases by Polyphenols.” Biochemical Pharmacology 63: 1165–1176. 10.1016/s0006-2952(02)00848-1 11931850

[61] Wu, Chuyan , Ke Wei , and Zhongli Jiang . 2017. “5α‐Reductase Activity in Women With Polycystic Ovary Syndrome: A Systematic Review and Meta‐Analysis.” Reproductive Biology and Endocrinology 15: 21. 10.1186/s12958-017-0242-9 28347315 PMC5369013

[62] Magoffin, Denis A . 2006. “Ovarian Enzyme Activities in Women With Polycystic Ovary Syndrome.” Fertility and Sterility 86: S9–S11. 10.1016/j.fertnstert.2006.03.015 16798289

[63] Yang, Fan , Chao Chen , Derang Ni , Yubo Yang , Jinhu Tian , Yuanyi Li , Shiguo Chen , Xingqian Ye , and Li Wang . 2023. “Effects of Fermentation on Bioactivity and the Composition of Polyphenols Contained in Polyphenol‐Rich Foods: A Review.” Foods 12: 3315. 10.3390/foods12173315 37685247 PMC10486714

[64] Wang, Mengyao , Lixuan Sang , and Siyu Sun . 2024. “Gut Microbiota and Female Health.” World J Gastroenterol 30: 1655–1662. 10.3748/wjg.v30.i12.1655 38617735 PMC11008377

[65] Teede, Helena J. , Chau Thien Tay , Joop J. E. Laven , Anuja Dokras , Lisa J. Moran , Terhi T. Piltonen , Michael F. Costello , et al 2023. “Recommendations From the 2023 International Evidence‐Based Guideline for the Assessment and Management of Polycystic Ovary Syndrome.” Journal of Clinical Endocrinology & Metabolism 108: 2447–2469. 10.1210/clinem/dgad463 37580314 PMC10505534

[66] Wen, Tao , Penghao Xie , Shengdie Yang , Guoqing Niu , Xiaoyu Liu , Zhexu Ding , Chao Xue , et al. 2022. “ggClusterNet: An R Package for Microbiome Network Analysis and Modularity‐Based Multiple Network Layouts.” iMeta 1: e32. 10.1002/imt2.32 38868720 PMC10989811

[67] Feng, Yinglin , Huimin Zheng , Chunhua Yin , Dong Liang , Siyou Zhang , Jingrui Chen , Feihong Mai , et al. 2024. “β‐resorcylic Acid Released by Limosilactobacillus Reuteri Protects Against Cisplatin‐Induced Ovarian Toxicity and Infertility.” Cell Reports Medicine 5: 101678. 10.1016/j.xcrm.2024.101678 39096912 PMC11384965

[68] Marwick, Ben , and Kalimuthu Krishnamoorthy . 2016. “cvequality: Tests for the Equality of Coefficients of Variation From Multiple Groups.” R Package Version 0.2.0. 10.32614/CRAN.package.cvequality

[69] Krishnamoorthy, K. , and Meesook Lee . 2013. “Improved Tests for the Equality of Normal Coefficients of Variation.” Computational Statistics 29: 215–232. 10.1007/s00180-013-0445-2

[70] Mangiola, Stefano , Ramyar Molania , Ruining Dong , Maria A. Doyle , and Anthony T. Papenfuss . 2021. “Tidybulk: An R Tidy Framework for Modular Transcriptomic Data Analysis.” Genome Biology 22: 42. 10.1186/s13059-020-02233-7 33482892 PMC7821481

[71] Love, Michael I. , Wolfgang Huber , and Simon Anders . 2014. “Moderated Estimation of Fold Change and Dispersion for RNA‐seq Data With DESeq. 2.” Genome Biology 15: 550. 10.1186/s13059-014-0550-8 25516281 PMC4302049

[72] Wu, Tianzhi , Erqiang Hu , Shuangbin Xu , Meijun Chen , Pingfan Guo , Zehan Dai , Tingze Feng , et al. 2021. “Clusterprofiler 4.0: A Universal Enrichment Tool for Interpreting Omics Data.” Innovation 2: 100141. 10.1016/j.xinn.2021.100141 34557778 PMC8454663

[73] Slenter, Denise N. , Martina Kutmon , Kristina Hanspers , Anders Riutta , Jacob Windsor , Nuno Nunes , Jonathan Mélius , et al. 2017. “WikiPathways: A Multifaceted Pathway Database Bridging Metabolomics to Other Omics Research.” Nucleic Acids Research 46: D661–D667. 10.1093/nar/gkx1064 PMC5753270 29136241

[74] Wickham, Hadley . 2011. “ggplot2.” WIREs Computational Statistics 3: 180–185. 10.1002/wics.147

[75] Yu, Guangchuang , David K. Smith , Huachen Zhu , Yi Guan , and Tommy Tsan‐Yuk Lam . 2017. “Ggtree: An R Package for Visualization and Annotation of Phylogenetic Trees With Their Covariates and Other Associated Data.” Methods in Ecology and Evolution 8: 28–36. 10.1111/2041-210X.12628

[76] Chen, Shifu . 2023. “Ultrafast One‐Pass FASTQ Data Preprocessing, Quality Control, and Deduplication Using Fastp.” iMeta 2: e107. 10.1002/imt2.107 38868435 PMC10989850

[77] Langmead, Ben , and Steven L. Salzberg . 2012. “Fast Gapped‐Read Alignment With Bowtie 2.” Nature Methods 9: 357–359. 10.1038/nmeth.1923 22388286 PMC3322381

[78] DeSantis, T. Z. , P. Hugenholtz , N. Larsen , M. Rojas , E. L. Brodie , K. Keller , T. Huber , et al. 2006. “Greengenes, a Chimera‐Checked 16S rRNA Gene Database and Workbench Compatible With ARB.” Appl Environ Microbiol 72: 5069–5072. 10.1128/aem.03006-05 16820507 PMC1489311

[79] Beghini, Francesco , Lauren J. McIver , Aitor Blanco‐Míguez , Leonard Dubois , Francesco Asnicar , Sagun Maharjan , Ana Mailyan , et al. 2021. “Integrating Taxonomic, Functional, and Strain‐Level Profiling of Diverse Microbial Communities With Biobakery 3.” Elife 10: e65088. 10.7554/eLife.65088 33944776 PMC8096432

[80] Xu, Shuangbin , Li Zhan , Wenli Tang , Qianwen Wang , Zehan Dai , Lang Zhou , Tingze Feng , et al. 2023. “MicrobiotaProcess: A Comprehensive R Package for Deep Mining Microbiome.” Innovation 4: 100388. 10.1016/j.xinn.2023.100388 36895758 PMC9988672

[81] Zhang, Yong , Tao Liu , Clifford A. Meyer , Jérôme Eeckhoute , David S. Johnson , Bradley E. Bernstein , Chad Nusbaum , et al. 2008. “Model‐Based Analysis of ChIP‐Seq (MACS).” Genome Biology 9: R137. 10.1186/gb-2008-9-9-r137 18798982 PMC2592715

