# Supplementary material for: Gut microbial‐derived 3,4‐dihydroxyphenylacetic acid ameliorates reproductive phenotype of polycystic ovary syndrome
Source: Imeta. 2025 Jul 15;4(5):e70065. doi: 10.1002/imt2.70065 (PMC12527991; doi:10.1002/imt2.70065)
Supplement: Supplementary file 1 — Figure S1: Metabolite composition of patients and mice with PCOS. Figure S2: DHPAA relates to reproductive system diseases and is decreased in PCOS. Figure S3: DHPAA alleviates PCOS‐like symptoms in LET‐induce PCOS‐like mice. Figure S4: Protective effects of DHPAA against PCOS links to inhibiting BMP signaling. Figure S5: The use of BMP signaling agonist abolishes protective effects of DHPAA against PCOS. Figure S6: The use of BMP signaling agonist abolishes protective effects of DHPAA against PCOS. Figure S7: DHPAA Production depends on the existence of gut microbiota. Figure S8: Streptococcus thermophilus is identified as a biomarker for PCOS and links to flavonoid degradation. Figure S9: β‐galactosidase activity is reduced in PCOS. [file IMT2-4-e70065-s001.docx]

# Supporting information to

# Gut microbial-derived 3,4-dihydroxyphenylacetic acid ameliorates reproductive phenotype of polycystic ovary syndrome

**Running title:** Microbial-derived DHPAA ameliorates PCOS

Pan Li^1,2,3#^, Li Xie^1#^, Huimin Zheng^1#^, Yinglin Feng^4#^, Feihong Mai^5^, Wenli Tang^1^, Jiajia Wang^1^, Zixin Lan^6^, Shuaijun Lv^1^, Thisun Jayawardana^3^, Sabrina Koentgen^3^, Shuangbin Xu^7^, Zhengwei Wan^8^, Yunjie Chen^9^, Haiyan Xu^2,10^, Sj Shen^3^, Fan Zhang^3^, Yuanhao Yang^11^, Georgina Hold^3^, Fangjie He^12^*, Emad M. El-Omar^3^*, Guangchuang Yu^7^*, and Xia Chen^1,2^*

^1^Ningbo Key Laboratory of Human Microbiome and Precision Medicine, Central Laboratory of the Medical Research Center, The First Affiliated Hospital of Ningbo University, Ningbo 315000, China

^2^Department of Obstetrics and Gynecology, The First Affiliated Hospital of Ningbo University, Ningbo 315000, China

^3^ UNSW Microbiome Research Centre, St George and Sutherland Clinical Campuses, UNSW Sydney 2217, Australia

^4^ Department of Obstetrics, Affiliated Foshan Maternity & Child Healthcare Hospital, Southern Medical University, Foshan 528000, China

^5^ Institute of Ecological Science, School of Life Science, South China Normal University, Guangzhou 510000, China

^6^ The Second Clinical Medical College, Southern Medical University, Guangzhou 510000, China

^7^ Department of Bioinformatics, School of Basic Medical Sciences, Southern Medical University, Guangzhou 510000, China

^8^ Department of Health Management & Institute of Health Management, Sichuan Provincial People's Hospital, University of Electronic Science and Technology of China, Chengdu 610000, China

^9^ Department of Pharmacy, The First Affiliated Hospital of Ningbo University, Ningbo 315000, China

^10^ Reproductive Medicine Center, The First Affiliated Hospital of Ningbo University, Ningbo 315000, China

^11^ Mater Research Institute, The University of Queensland, Woolloongabba, Queensland 4102, Australia

^12^Fujian Maternity and Child Health Hospital, College of Clinical Medicine for Obstetrics & Gynecology and Pediatrics, Fujian Medical University, Fuzhou 350000, China.

# These authors contributed equally: Pan Li, Li Xie, Huimin Zheng, Yinglin Feng.

* Correspondence: dr_hefangjie@163.com (Fangjie He), e.el-omar@unsw.edu.au (Emad El-Omar), gcyu1@smu.edu.cn (Guangchuang Yu), and chenx_fsyyy@163.com (Xia Chen)


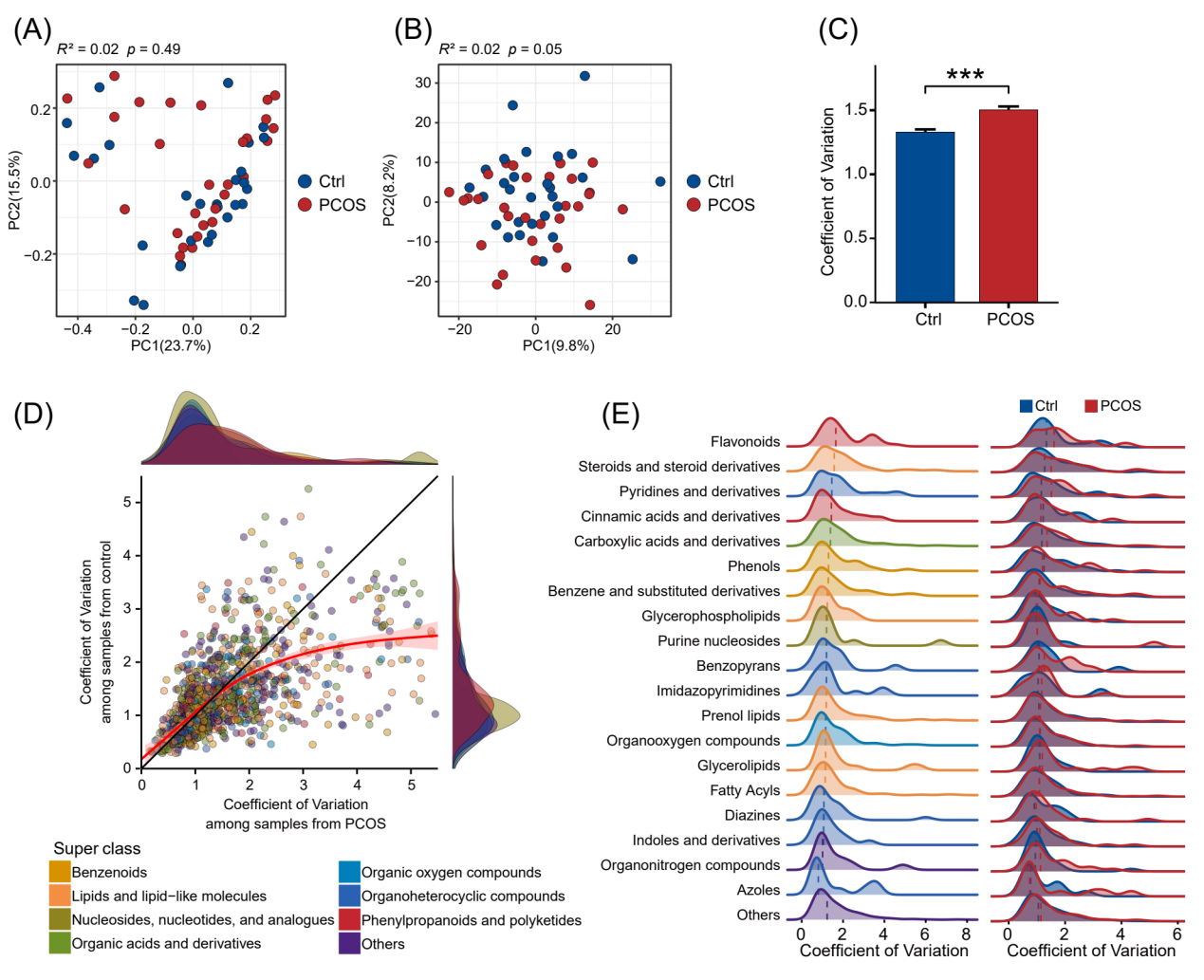


## **Figure S1 Metabolite composition of patients and mice with PCOS.** (A and B) Principal coordinate analysis (PCoA) based on Bray-Curtis (A) and Euclidean (B) distances. Each point represents a single sample, coloured by group. Differences in beta diversity between PCOS and Ctrl were tested by permutational multivariate analysis of variance (PERMANOVA). (C) The overall coefficient of variation (CV) between patients with PCOS and healthy controls. Data are presented as the mean ± SEM; ****p* < 0.001 according to two-tailed Wilcoxon rank-sum test. (D) The CV for each metabolite in PCOS and controls. Each point represents one metabolite; the red line is the fitted curve of points; the black line represents equal CV between patients with PCOS and healthy controls. (E) The distribution of CV among different classes.


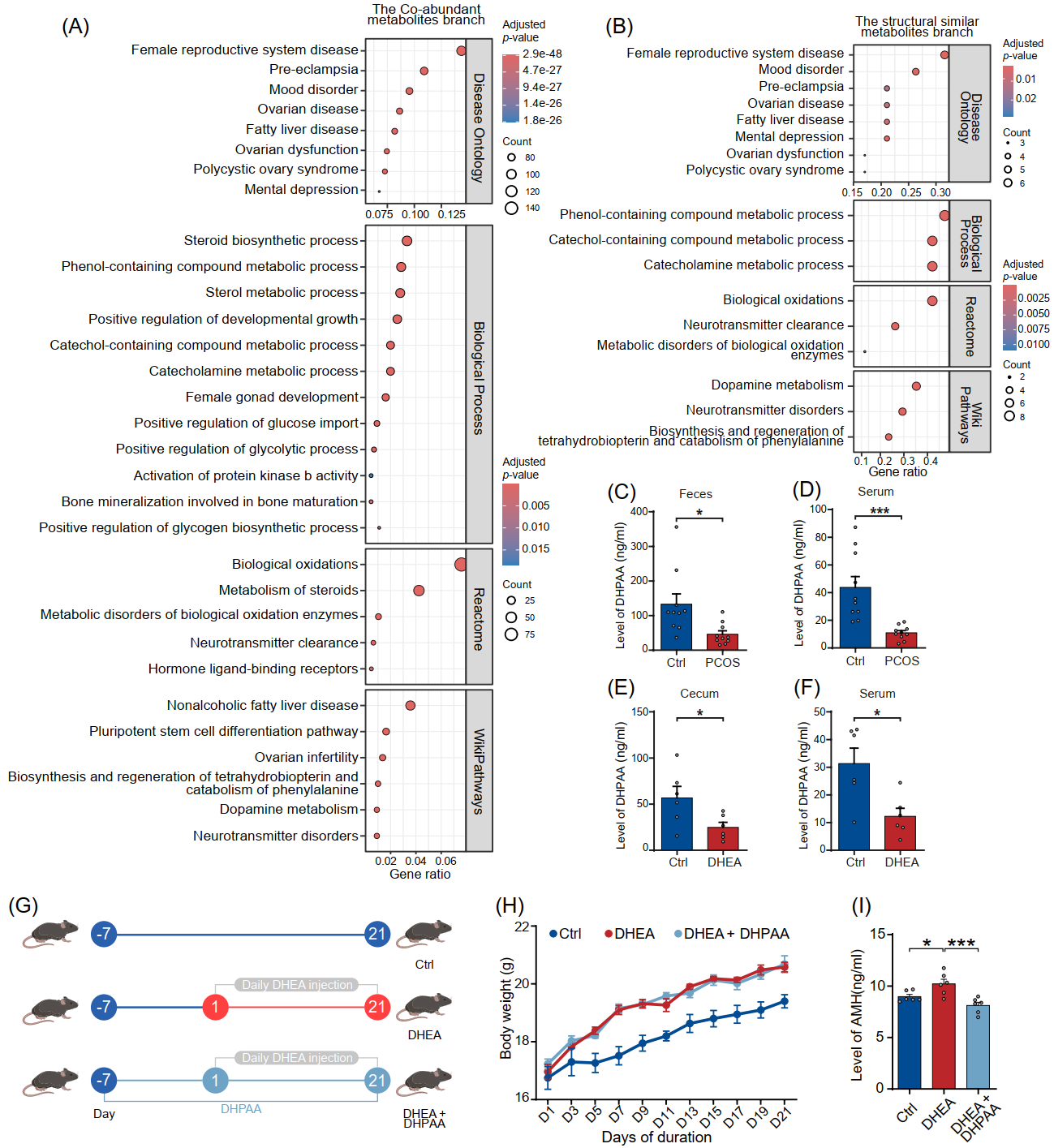


**Figure S2 DHPAA relates to reproductive system diseases and is decreased in PCOS.** (A) Enrichment analysis of metabolites showed co-abundant with DHPAA based on Disease Ontology, Gene ontology, Reactome, and WikiPathways databases. (B) Enrichment analysis of metabolites with similar structure to DHPAA based on Disease Ontology, Gene ontology, Reactome, and WikiPathways databases. (C-D) The concentration of DHPAA in the feces (C) and serum (D) of healthy control and patients with PCOS. (E-F) The concentration of DHPAA in the cecum (E) and serum (F) of control mice and PCOS-like mice (*n* = 6 mice per group). (G) Schematic representation and timeline for Ctrl, PCOS-like mice, and PCOS-like mice pre-treated with DHPAA. (H) Body weight of mice in DHPAA treatment experiment. (I) Expression levels of *Amh* in ovary (*n* = 6 mice per group). The dose of DHPAA is 80 μM. Data are presented as the mean ± SEM. **p* < 0.05, ****p* < 0.001; two-tailed Student’s *t* test in C, D, E, F; two-tailed one-way ANOVA following Dunnett’s multiple comparisons test.


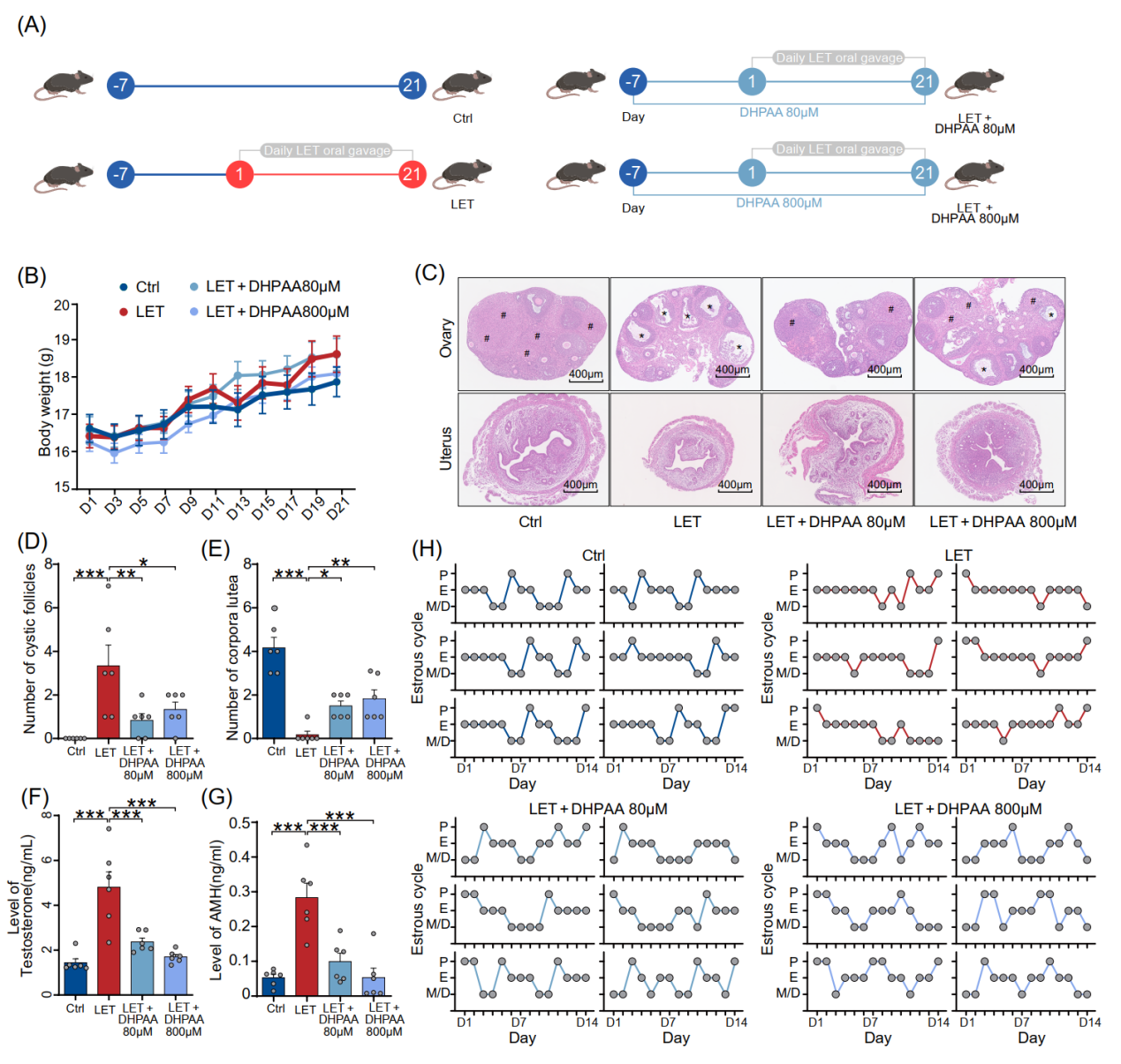


**Figure S3 DHPAA alleviates PCOS-like symptoms in LET-induce PCOS-like mice.** (A) Schematic representation and timeline for Ctrl, PCOS-like mice, and PCOS-like mice pre-treated with DHPAA (80 μM and 800 μM). (B) Body weight of mice in DHPAA treatment experiment. (C) Hematoxylin and eosin (H&E) staining of representative mouse ovaries and uterus from control mice, PCOS-like mice, PCOS-like mice with 80 μM DHPAA treatment, and PCOS-like mice with 800μM DHPAA treatment (scale bars, 400 μm). The corpora lutea are indicated by # and the cystic follicle is indicated by *. (D) Quantitative analysis of cystic follicles in the ovaries (*n* = 6 mice per group). (E) Quantitative analysis of corpora lutea in the ovaries (*n* = 6 mice per group). (F) Levels of testosterone in serum (*n* = 6 mice per group). (G) Levels of AMH in serum (*n* = 6 mice per group). (H) Estrous cycle analysis (*n*  =  6 mice per group). Data are presented as the mean ± SEM. **p* < 0.05, ****p* < 0.001; two-tailed Student’s *t* test or two-tailed one-way ANOVA following the Dunnett’s multiple comparisons test.


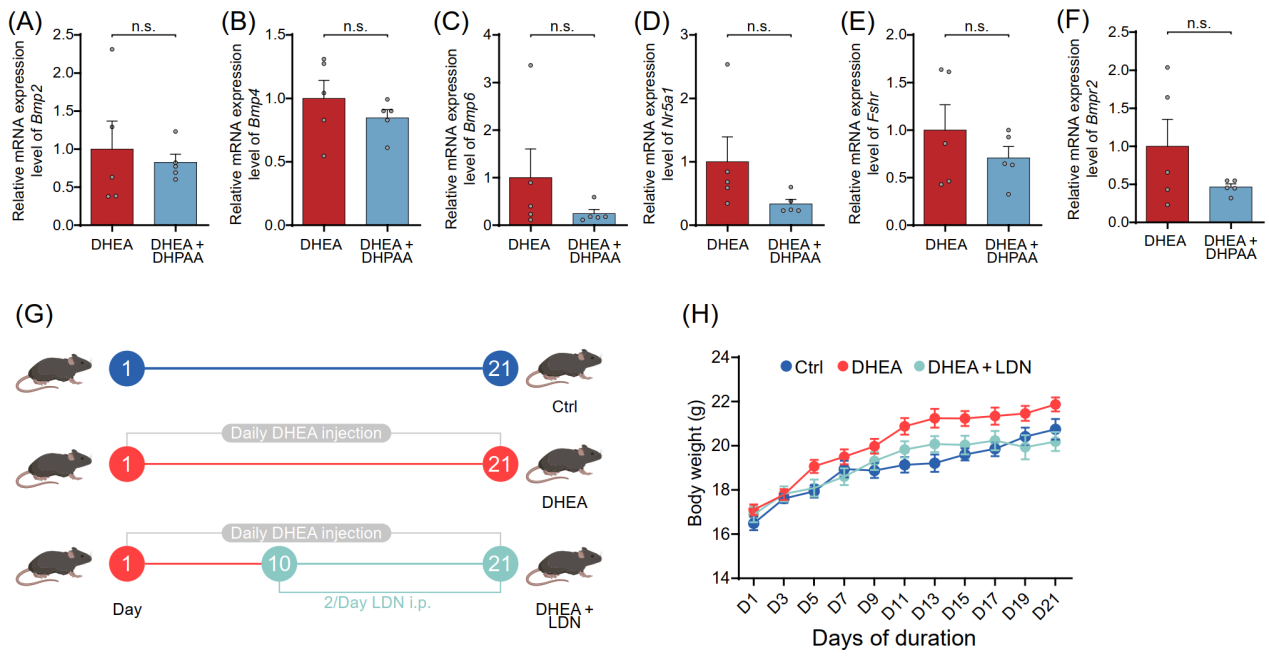


## **Figure S4 Protective effects of DHPAA against PCOS links to inhibiting BMP signaling.** (A-G) Expression levels of *Bmp2*, *Bmp4*, *Bmp6*, *Nr5a1*, *Fshr*, and *Bmpr2* in ovary (*n* = 5 mice per group). (G) Schematic representation and timeline for control mice, PCOS-like mice and PCOS-like mice treated with LDN193189. (H) Body weight of mice. Data are presented as the mean ± SEM. **p* < 0.05, ****p* < 0.001; two-tailed Student’s *t* test in A-F.


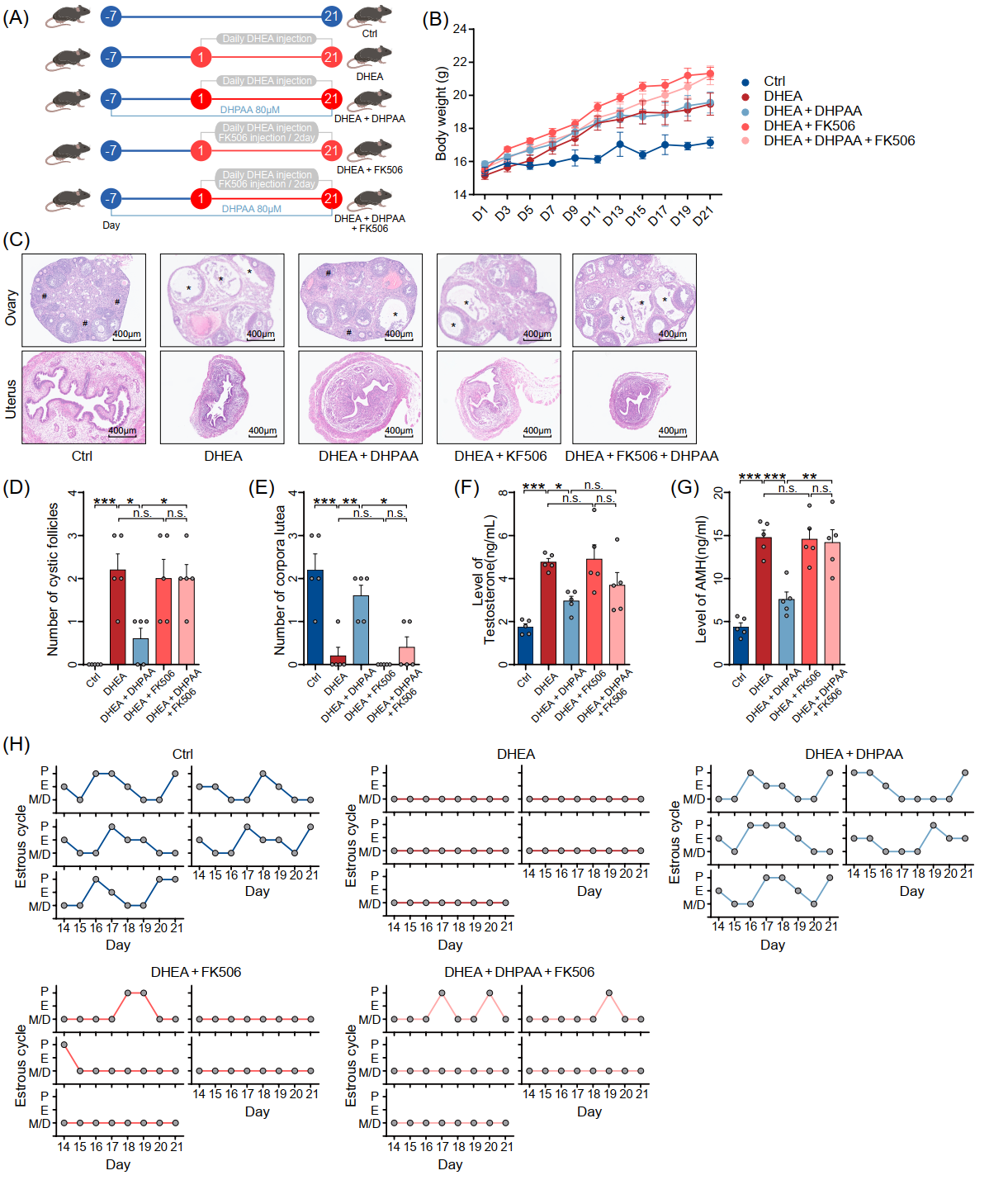


**Figure S5 The use of BMP signaling agonist abolishes protective effects of DHPAA against PCOS.** (A) Schematic representation and timeline for control mice, PCOS-like mice, PCOS-like mice treated with DHPAA, PCOS-like mice treated with FK506, and PCOS-like mice treated with DHPAA and FK506. (B) The body weight of mice. (C) Hematoxylin and eosin (H&E) staining of representative mouse ovaries and uterus from control mice, PCOS-like mice, PCOS-like mice with 80μM DHPAA treatment, PCOS-like mice with FK506 treatment, and PCOS-like mice with 80μM DHPAA and FK506 treatment (scale bars, 400 μm). The corpora lutea are indicated by # and the cystic follicle is indicated by *. (D) Quantitative analysis of cystic follicles in the ovaries (*n* = 5 mice per group). (E) Quantitative analysis of corpora lutea in the ovaries (*n* = 5 mice per group). (F) Levels of testosterone in serum (*n* = 5 mice per group). (G) Levels of AMH in serum (*n* = 5 mice per group). (H) Estrous cycle analysis (*n*  = 5 mice per group). Data are presented as the mean ± SEM. **p* < 0.05, ***p* < 0.01, ****p* < 0.001; two-tailed Student’s *t* test or two-tailed one-way ANOVA following the Tukey multiple comparisons test.


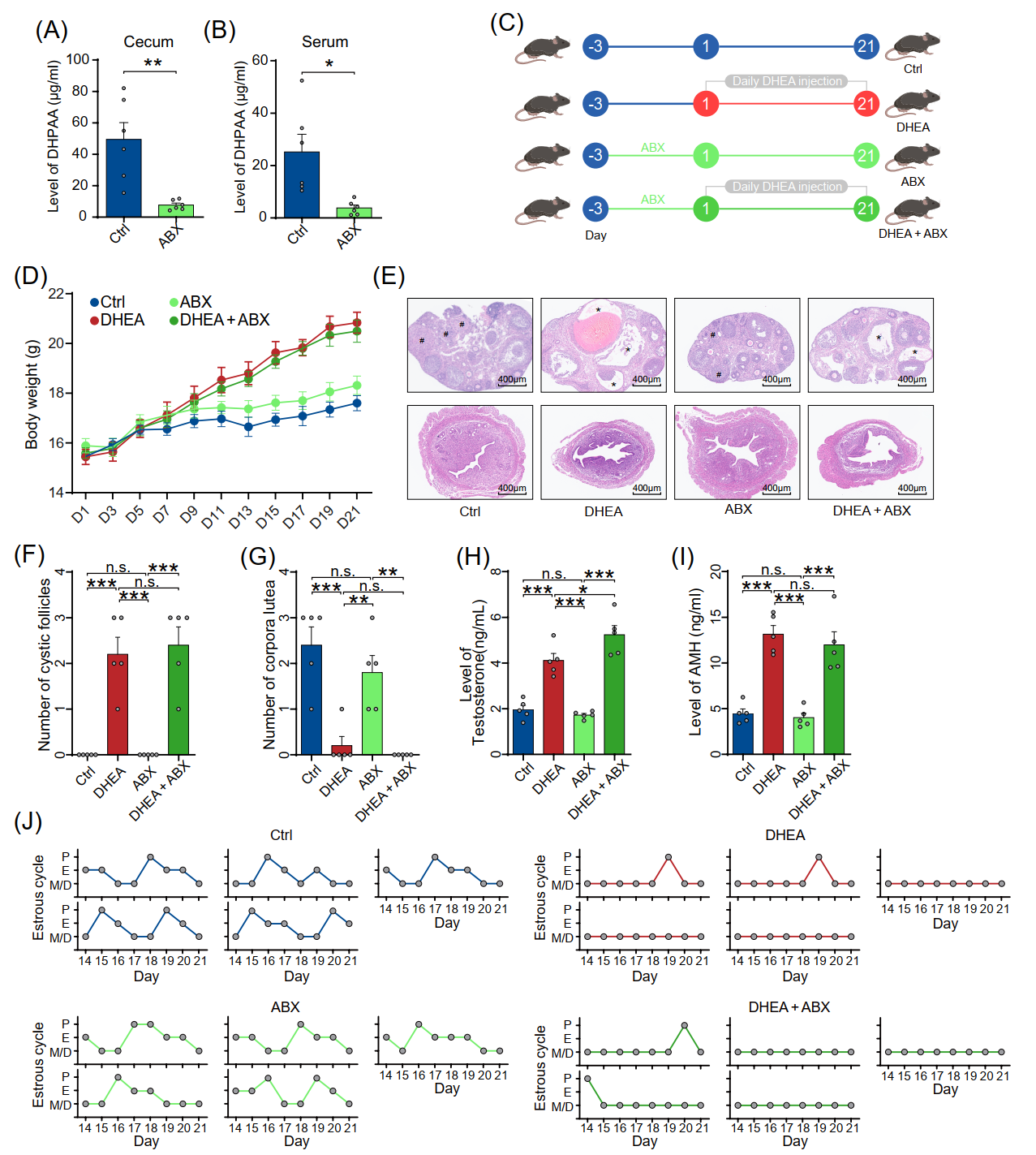


**Figure S6 The use of BMP signaling agonist abolishes protective effects of DHPAA against PCOS.** (A-B) The concentration of DHPAA in the cecum (A) and serum (B) of mice treated with or without antibiotics (*n* = 6 mice per group). (C) Schematic representation and timeline for control mice, PCOS-like mice, mice treated with antibiotic cocktail, and PCOS-like mice treated with antibiotic cocktail. (D) The body weight of mice. (E) Hematoxylin and eosin (H&E) staining of representative mouse ovaries and uterus from control mice, PCOS-like mice, mice treated with antibiotic cocktail, and PCOS-like mice treated with antibiotic cocktail (scale bars, 400 μm). The corpora lutea are indicated by # and the cystic follicle is indicated by *. (F) Quantitative analysis of cystic follicles in the ovaries (*n* = 5 mice per group). (G) Quantitative analysis of corpora lutea in the ovaries (*n* = 5 mice per group). (H) Levels of testosterone in serum (*n* = 5 mice per group). (I) Levels of AMH in serum (*n* = 5 mice per group). (J) Estrous cycle analysis (*n*  = 5 mice per group). Data are presented as the mean ± SEM. **p* < 0.05, ***p* < 0.01, ****p* < 0.001; two-tailed Student’s *t* test or two-tailed one-way ANOVA following the Tukey multiple comparisons test.


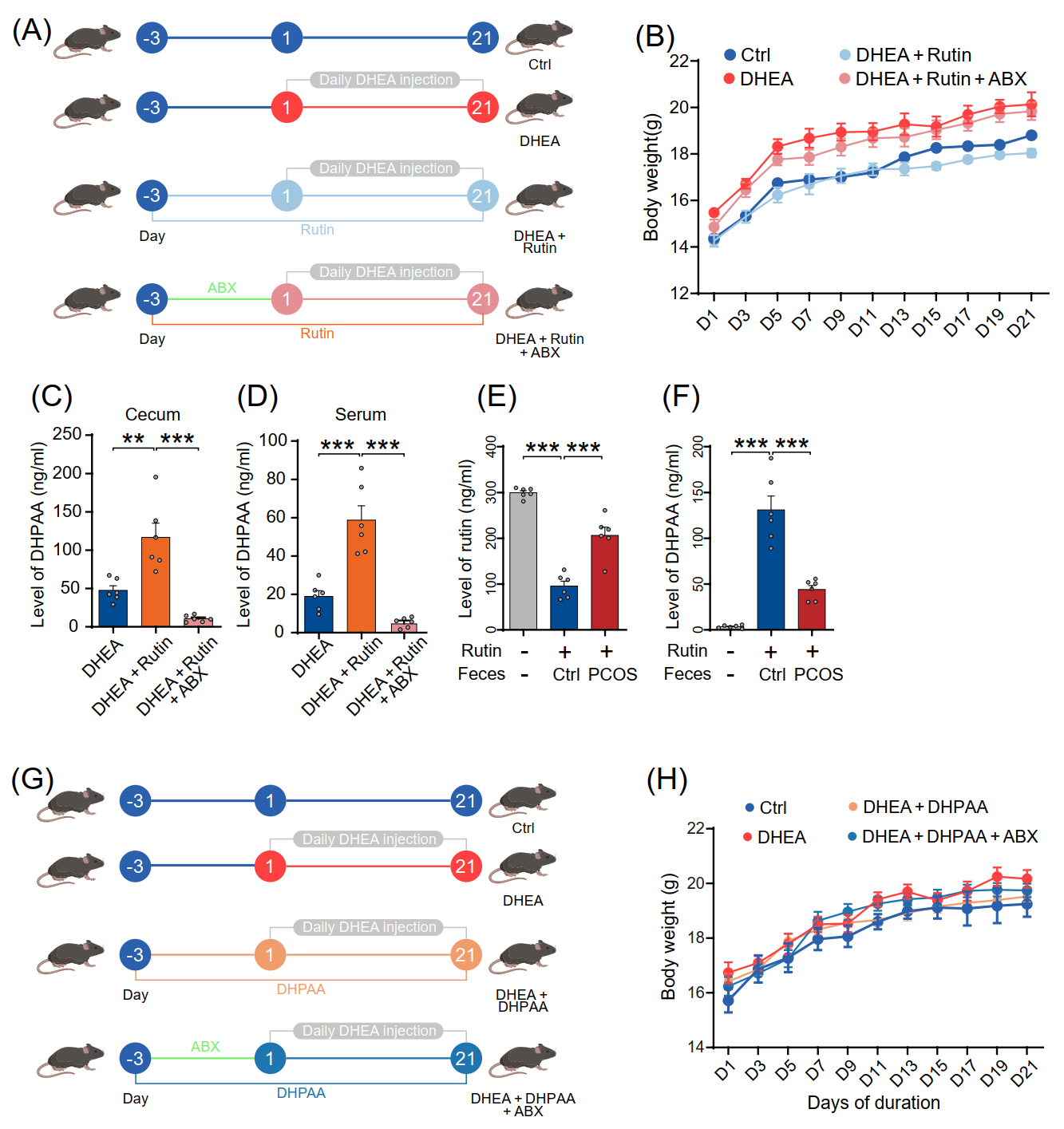


**Figure S7 DHPAA Production depends on the existence of gut microbiota.** (A) Schematic representation and timeline for control mice, PCOS-like mice, PCOS-like mice treated with rutin, and antibiotics-pre-treated PCOS-like mice treated with rutin. (B) Body weight of mice. (C-D) The concentration of DHPAA in the cecum (C) and serum (D) of PCOS-like mice, PCOS-like mice treated with rutin, and antibiotics-pre-treated PCOS-like mice treated with rutin (*n* = 6 mice per group). (E) The concentration of rutin in the culture supernatant of fecal samples from healthy controls and patients with PCOS with rutin. (F) The concentration of DHPAA in the culture supernatant of fecal samples from healthy controls and patients with PCOS with rutin. (G) Schematic representation and timeline for control mice, PCOS-like mice, PCOS-like mice treated with DHPAA, and antibiotics-pre-treated PCOS-like mice treated with DHPAA. (H) Body weight of mice. The dose of rutin is 50 μM. Data are presented as the mean ± SEM. **p* < 0.05, ***p* < 0.01, ****p* < 0.001; two-tailed Student’s *t* test or two-tailed one-way ANOVA following Dunnett’s multiple comparisons test.


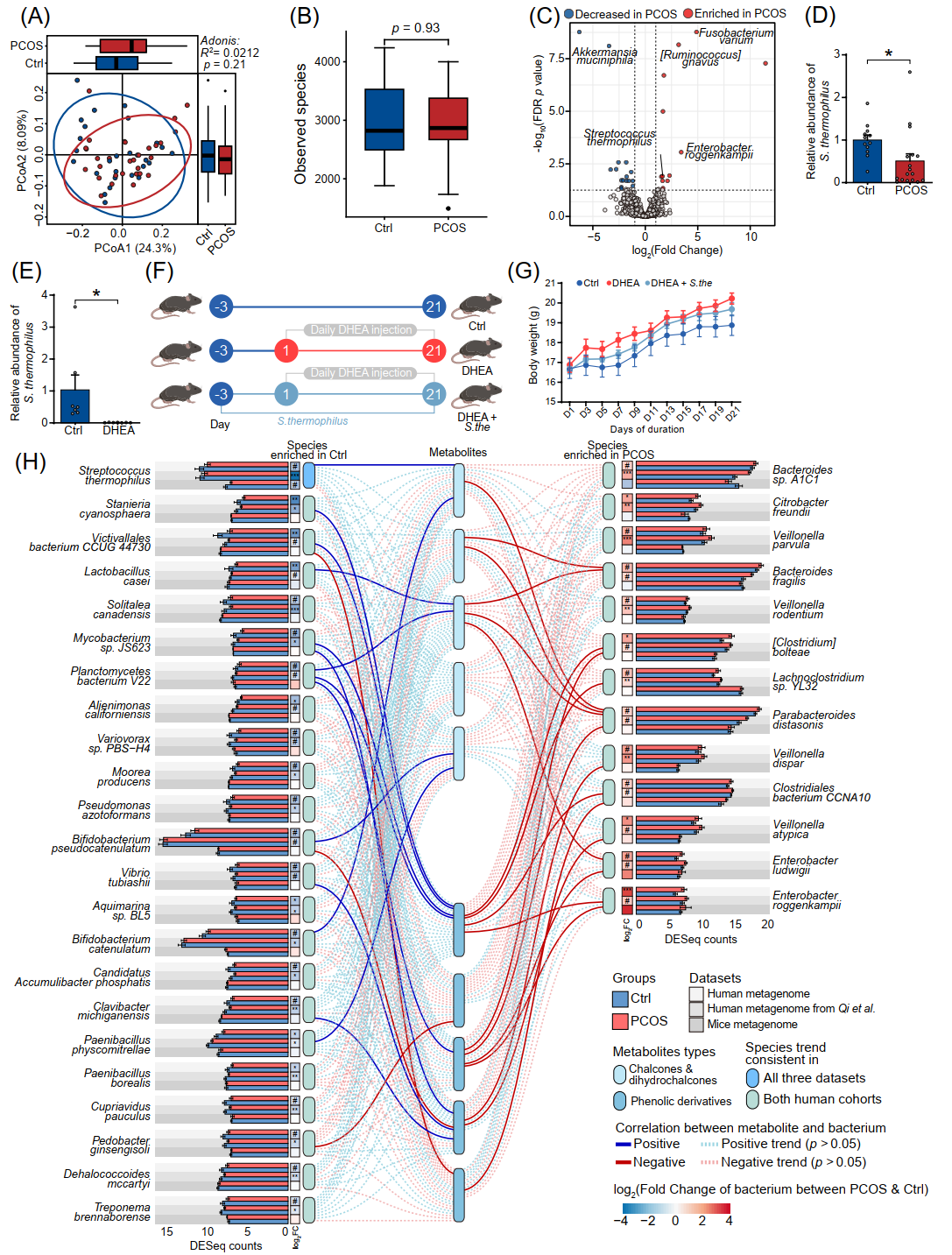


**Figure S8 Streptococcus thermophilus is identified as a biomarker for PCOS and links to flavonoid degradation.** (A) Principal coordinate analysis (PCoA) based on Bray-Curtis distances. Each point represents a single sample, coloured by group. Differences in beta diversity between PCOS and Ctrl were tested by permutational multivariate analysis of variance (PERMANOVA). (B) Observed species between patients with PCOS and healthy controls (two-tailed Student’s *t* test). (C) Volcano plots of the differential bacterial species. The x and y axis are using the log2 Fold change between PCOS and control group and FDR *p* value by DeSeq2, respectively. (D) Relative abundance of *S. thermophilus* in feces from healthy controls and patients with PCOS (two-tailed Student’s *t* test). (E) Relative abundance of *S. thermophilus* in feces from control mice and PCOS-like mice (two-tailed Student’s *t* test). (F) Schematic representation and timeline for control mice, PCOS-like mice, PCOS-like mice pre-treated with *S. thermophilus*. (G) Body weight of mice. (H) The correlation between differential species and bacterial flavonoid derivatives.The barplots show the DESeq counts of bacterial species in human cohort, mice experiments, and independent human cohort. The lines represent the correlation between bacteria and bacterial flavonoid downstream derivatives while blue and red represent positive and negative correlation respectively. Solid line and dashed line indicate whether *P*-value is below or above 0.05. The correlation analysis is conducted using *Spearman* method. The log_2_(Fold change) represents the fold change of the abundance of bacterium between PCOS and control group.


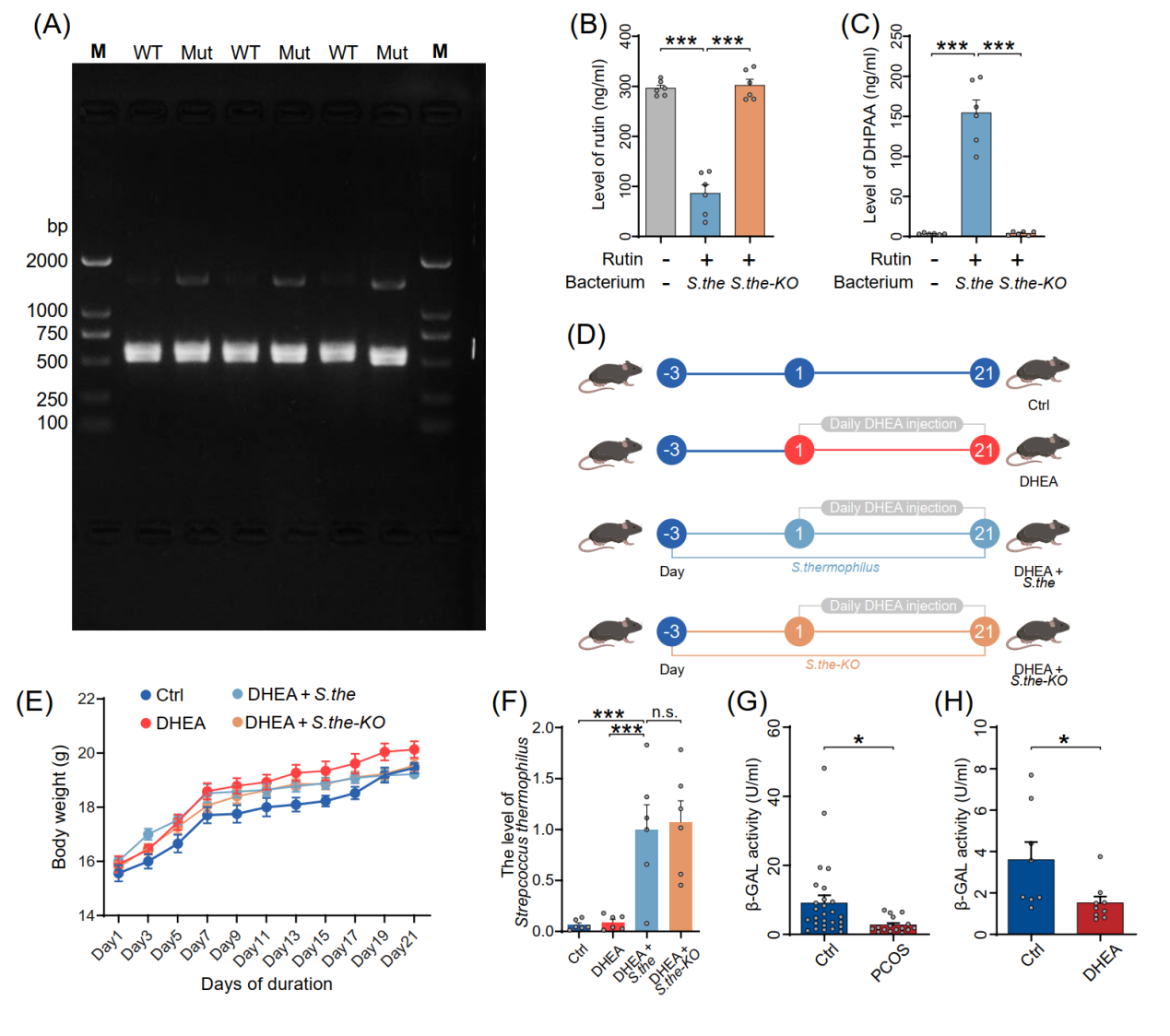


## **Figure S9 β-galactosidase activity is reduced in PCOS.** (A) PCR was performed to prove the successful construction of mutant *S. thermophilus* strain. (B) The concentration of rutin in the culture supernatant of fecal samples from mice treated with wild type strain or KO strain with rutin. (C) The concentration of DHPAA in the culture supernatant of fecal samples from mice treated with wild type strain or KO strain with rutin. (D) Schematic representation and timeline for control mice, PCOS-like mice, PCOS-like mice pre-treated with wild type *S. thermophilus*, and PCOS-like mice pre-treated with β-GAL-mutant *S. thermophilus*. (E) Body weight of mice. (F) Relative abundance of *S. thermophilus* in feces of mice. (G) β-galactosidase activities in the fecal samples from healthy controls and patients with PCOS (H) β-galactosidase activities in the fecal samples from control mice and PCOS-like mice. Data are presented as the mean ± SEM. **p* < 0.05; two-tailed Student’s *t* test.
